# Psychological and Behavioral Insights From Social Media Users: Natural Language Processing–Based Quantitative Study on Mental Well-Being

**DOI:** 10.2196/60286

**Published:** 2025-01-20

**Authors:** Xingwei Yang, Guang Li

**Affiliations:** 1 Information Technology Management Ted Rogers School of Management Toronto Metropolitan University Toronto, ON Canada; 2 Smith School of Business Queen's University Kingston, ON Canada

**Keywords:** social media, natural language processing, social interaction, decision support system, depression detection

## Abstract

**Background:**

Depression significantly impacts an individual’s thoughts, emotions, behaviors, and moods; this prevalent mental health condition affects millions globally. Traditional approaches to detecting and treating depression rely on questionnaires and personal interviews, which can be time consuming and potentially inefficient. As social media has permanently shifted the pattern of our daily communications, social media postings can offer new perspectives in understanding mental illness in individuals because they provide an unbiased exploration of their language use and behavioral patterns.

**Objective:**

This study aimed to develop and evaluate a methodological language framework that integrates psychological patterns, contextual information, and social interactions using natural language processing and machine learning techniques. The goal was to enhance intelligent decision-making for detecting depression at the user level.

**Methods:**

We extracted language patterns via natural language processing approaches that facilitate understanding contextual and psychological factors, such as affective patterns and personality traits linked with depression. Then, we extracted social interaction influence features. The resultant social interaction influence that users have within their online social group is derived based on users’ emotions, psychological states, and context of communication extracted from status updates and the social network structure. We empirically evaluated the effectiveness of our framework by applying machine learning models to detect depression, reporting accuracy, recall, precision, and *F*_1_-score using social media status updates from 1047 users along with their associated depression diagnosis questionnaire scores. These datasets also include user postings, network connections, and personality responses.

**Results:**

The proposed framework demonstrates accurate and effective detection of depression, improving performance compared to traditional baselines with an average improvement of 6% in accuracy and 10% in *F*_1_-score. It also shows competitive performance relative to state-of-the-art models. The inclusion of social interaction features demonstrates strong performance. By using all influence features (affective influence features, contextual influence features, and personality influence features), the model achieved an accuracy of 77% and a precision of 80%. Using affective features and affective influence features also showed strong performance, achieving 81% precision and an *F*_1_-score of 79%.

**Conclusions:**

The developed framework offers practical applications, such as accelerating hospital diagnoses, improving prediction accuracy, facilitating timely referrals, and providing actionable insights for early interventions in mental health treatment plans.

## Introduction

Depression is characterized by a constant depressed mood and the inability to anticipate happiness or pleasure; [1] it has emerged as one of the major challenges globally [2]. Approximately 350 million people experience depression worldwide [3]. Without timely psychiatric interventions, a patient may have worsening symptoms, leading to more severe consequences such as self-harm, physical disability, or even suicide [4]. This problem has been exacerbated by the COVID-19 pandemic. Fear of COVID-19 infection, lack of social support, isolation, and limited access to medical resources during the COVID-19 pandemic have increased psychological distress [5,6]. Compared to the prepandemic period, depression rates almost doubled in 2020 to 2021 [7]. Despite its severity and seriousness, depression remains significantly underdiagnosed and undertreated [8]. Thus, there is an increasing need for intelligent clinical decision support tools to offer timely depression diagnoses and referral services so that people can receive early intervention or treatment before developing more serious mental health conditions.

Traditional methods of depression detection and treatment are based on questionnaires and personal interviews, which usually require long one-on-one physical engagements where psychiatrists gather information from patients and then recommend medications or treatments. This process can be both time consuming and costly, not only because of its complexity but also because of the expenses associated with the required trained personnel [9]. Thus, it is challenging for mental health professionals to offer immediate detection, referral services, proactive interventions, or treatments for those at risk and prepare for the next public health emergency, such as COVID-19, particularly when in-person assessments are difficult or costly. This challenge has motivated the exploration of more cost-effective and efficient methods, such as leveraging data from social media [10]. Platforms such as Meta are commonly used to share feelings and emotions. This makes social media a new venue for depression investigations [11-13] because they provide an unbiased exploration of language use and behavioral patterns of individuals [14]. Moreover, research has shown that depression can affect how individuals express themselves in their social media posts [15]. Therefore, there is an increasing trend toward using abundant text data and information from social media to examine individuals’ sentiments and behaviors in mental health studies [16,17]. However, manually analyzing texts is not practical for timely psychiatric support due to the exponential growth of text data on social media and the increasing number of cases of mental illnesses. Thus, there is a rising need to use machine learning (ML) and natural language processing (NLP) techniques to deal with the large volume of data. Existing research in this domain primarily focuses on finding the key indicators, such as linguistic and sentiment features, to enhance the accuracy of depression detection [18,19]. For example, many studies focus on extracting textual content from social media data using linguistic features such as semantics and syntactic features [20], a bag of words [21], and n-grams [22]. Although these features can help yield reasonable results in depression detection, they overlook several critical aspects.

First, these linguistic features do not thoroughly reflect an individual’s psychological patterns and states. Affective patterns (affect refers to emotions that change or influence the behaviors or thoughts of an individual) in language expressions are important dimensions of psychological patterns. They can reveal an individual’s moods, sentiments, and emotional factors beyond simple linguistic features for analyzing mental disorders on social media. Studies show that frequent negative affect expression has higher connections with depressive states, while positive affect tends to have a lower connection to depression [23-25]. Thus, emotions play an essential role in diagnosing mental illnesses such as depression [11]. In addition, an individual’s psychological states are directly related to depression. Personality has been used to distinguish social media users’ activities, status updates content, and language use habits [26,27]. Moreover, Klein et al [28] and Hakulinen et al [29] identified the links between depression and personality types and found that certain personality traits may serve as predictive factors and influence the treatment of depression. Second, contextual information about the words is another critical dimension. Because pretrained language models have shown strong contextual modeling ability, they are used in understanding texts in various applications [30,31]. By considering the sequence of all words in the documents, sequence-level semantics can be learned to reflect the diverse contexts and meanings of words in sentences. For example, in the sentence “My car was left on the left-hand side,” the word “left” is used 2 times, but it carries 2 different meanings. The first “left” is a verb suggesting the action performed on the car, while the second “left” is a noun specifying the car’s location. The contextual meaning (eg, the meaning derived from the words’ surroundings) can vary for the same word. Thus, contextual information is also a crucial aspect of understanding individuals’ mental states.

Third, existing research often overlooks the impact of social interaction*s* when analyzing features for mental disorders on social media. Social interactions reflect how users influence each other within an online social group. According to social influence theory, individuals in social environments significantly shape each other’s attitudes, beliefs, and actions [32]. Recent psychological studies show that family, friends, or colleagues can directly or indirectly affect mental well-being [33]. For instance, in internet-based communities, recreational discussions, or chat groups, a negative mood can spread among different individuals [34,35]. Mental illnesses are influenced by an individual’s daily social interactions [36]. This impact can be even more substantial on social media, where individuals are influenced by their friends’ shared conventions and norms. Therefore, investigating the influence of social interaction is crucial for monitoring psychological patterns and states on social media. Studies by Murarka et al [37], Cui et al [38], and Ghosal and Jain [39] analyzed emotions, personality, and pretrained models for mental health problems, but they did not account for the impact of social interactions. To study the mental well-being of social media users, an individual’s mental health is reflected by more than just the content of their status updates. Thus, in our study, we not only account for psychological patterns and states and contextual information but also incorporate the influence of social interactions by considering the social network structure within these feature patterns.

This study aimed to offer a novel methodological language framework to enhance intelligent decision-making in the mental health domain. Our contributions are outlined as follows:

- We developed a unified language framework using NLP and ML techniques to enhance the detection of depression at the user level, making it more effective and accurate.
- Our framework advances previous studies by extracting and analyzing language patterns to comprehensively understand the contextual and psychological features (eg, affective patterns and personality) associated with depressive symptoms.
- We extracted features of social interaction influence, capturing how users influence their online social group. These features consider users’ emotions, psychological states, and communication context from status updates and social network structure, differentiating individuals with depression from those without.

Our study aims for early-stage detection and initial screening to identify individuals at risk of getting depression and narrow down those needing closer observation or follow-up. It is designed to complement, not replace, clinical diagnoses, offering insights that support timely interventions and enhance patient care. Our framework can enrich the reliability and knowledge base of decision support systems for health care providers, enabling more precise diagnoses and timely referrals, interventions, and treatment plans. The framework is designed to be adaptable, enabling researchers to apply similar methods to their own datasets and explore psychological, contextual, and social influence factors. While it provides valuable insights, the framework is not intended to draw definitive conclusions but rather to offer a structured approach to understanding social interactions and their impact. Beyond its focus on mental illness, it can be extended to other domains, generating interpretable findings tailored to specific contexts.

The paper is organized as follows. In the Methods section, we introduce our proposed framework and present the language feature discovery processes and the influence analysis. In the Results section, we empirically evaluate the effectiveness of our framework. We discuss the implications of our results, suggest directions for future research, and conclude the paper in the Discussion section.

## Methods

### Psychological and Contextual Analysis NLP Framework

**Figure 1:**
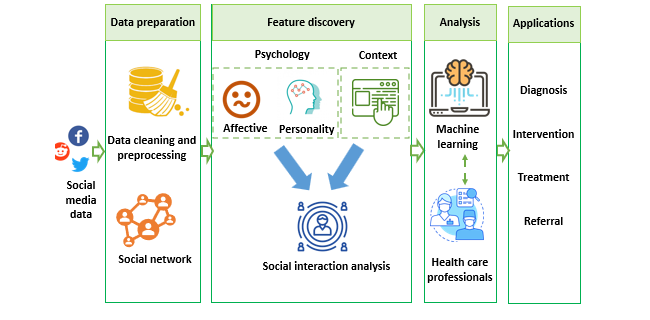
The proposed psychological and contextual analysis natural language processing framework. It starts with extensive data preparation and cleaning procedures and establishes a user-friendship network based on users’ social connections on social media. We then apply machine learning models to effectively manage high-volume and high-speed user-generated data to analyze the depression states of users within social media environments. This framework can be used in various applications.

This section presents our methods for inferring language patterns and conducting an analysis of social interactions from social media content. Figure 1 shows a conceptual representation of the proposed psychological and contextual analysis NLP framework to extract affective, personality, contextual, and social interaction influence features. Given the data collected from social media platforms, our framework starts with extensive data preparation and cleaning procedures. In addition, we establish a user-friendship network based on users’ social connections on social media. This network shows if one user is connected to others (ie, if this user is a friend of other users).

In the feature discovery phase, our framework deduces depressive behaviors from social media users, considering both contextual and psychological perspectives. We first focus on extracting features, including (1) affective features—these suggest users’ psychological patterns such as mood, sentiment, and emotional factors as expressed in their social media postings, (2) personality features—these reveal how users’ psychological states (ie, personalities) affect depression states, and (3) contextual features—these show sequence-level semantics learned from users’ social media postings to reflect different contexts and meanings of each word within sentences by considering the sequence of all words. On the basis of these features, we proceeded to perform a social interaction analysis to understand how the interactions between individuals with and without depression influence each other within their user-friendship network. We considered both the social network structure and users’ psychological and contextual features for extracting social interaction influence features, where the structure of the user-friendship network shows the strength of connections among users in the social media environment. Then, we applied ML models to effectively manage high-volume and high-speed user-generated data to analyze the depression states of users within social media environments.

### Data Cleaning and Preparation

We illustrated and evaluated our proposed framework using datasets from the study by Kosinski et al [40], which contain 197,230 Facebook (Meta Platforms, Inc) status updates from 1047 users, along with their associated Center for Epidemiologic Studies Depression (CES-D) Scale questionnaire scores. These datasets also include user postings, network connections, and personality responses. Note that datasets from other general social networks or online discussion forums can also be used. To minimize the privacy impact regarding using text data, we followed privacy-preserving procedures [41] to ensure anonymity throughout our analysis.

We obtained status updates from all users, regardless of their demographics, as our focus was on a general observation of social network users. Each user’s status updates were then aggregated based on their anonymous IDs. We cleaned and preprocessed the data to ensure consistent content. Text preprocessing was conducted in Python (Python Software Foundation), involving correcting spelling errors with SpellChecker (package in Python), converting text to lowercase, removing special characters and numbers, and expanding contractions. We used the NLTK package (Natural Language Toolkit, a Python library for NLP) to remove stop words such as “the,” “is,” and “our,” which are the most common words that appear in a sentence and do not contribute to its meaning. Thus, we removed stop words without sacrificing the meaning of the sentence. Our analysis assesses users’ personality features based on the Big Five Factor Model using their responses to the International Personality Item Pool (IPIP) [42]. We determined users’ depression levels by obtaining their responses to the CES-D Scale questionnaire, which is a commonly used approach to diagnose depression [43]. It is a 20-item screening questionnaire that captures users’ responses to questions related to depressive states. These questions assess the frequency of feelings and behaviors over the past week, including loneliness, sadness, and restless sleep. The higher the score, the more likely an individual is to experience depression. We adopted a similar threshold of 25, as suggested by Lewinsohn et al [44] and Julian et al [45], to label users with depression (total CES-D score >25) and without depression.

We begin by extracting affective, personality, and contextual features from status updates, followed by deriving social interaction influence features based on social network connections. The subsequent sections detail the procedure to identify psychological (eg, affective and personality) and contextual features. These features provide insights into how emotional states, personality traits, and contextual factors interact with social influences. By analyzing these features, we can better understand the dynamics of social interactions and their broader implications for applications such as behavior modeling and targeted interventions.

### Discover Affective Features

This section outlines how we extracted affective features. Emotions are essential in diagnosing mental illnesses [46]. Several studies show that depressive symptoms are more related to negative affect, while individuals without depression are more related to positive affect [23,24]. In our study, we extracted affective features to reveal individuals’ emotional states and understand users’ moods, sentiments, and emotional factors beyond simple linguistic features. Multimedia Appendix 1 shows the process of extracting emotion intensity features and emotion word features to represent the affective features. Because different words can convey affect to various degrees (eg, intensities), we used the NRC Emotion Intensity Lexicon [47] to extract the emotion words from users’ social media postings. The lexicon includes >10,000 words related to emotions, including surprise, anger, joy, fear, trust, sadness, anticipation, and disgust, with different intensities. Both common English words and prominent words in social media are covered. For each word *t*, the intensity of a particular emotion *e* falls within the range of 0 to 1. A value of “1” indicates the highest intensity of a particular emotion *e* conveyed by the word *t*, while “0” indicates the lowest intensity of emotion *e* for the word *t*, where *e* ∈ {fear, sadness, anger, joy, anticipation, trust, disgust, and surprise}. Thus, each emotion word has an emotion intensity vector *v*(*t*) showing the intensities of 8 emotion dimensions. We first calculated how often each emotion word appears within individuals’ social media postings to obtain an affective feature vector from a user’s posting. Then, for each emotion word *t*, we multiplied the emotion intensity vector *v*(*t*) with the frequency. After that, we computed the average intensity for all emotion words to obtain the affective feature vector.

For example, a user uses 3 emotion words “threat,” “illness,” and “sunny” in social media postings with a frequency of *freq*(*threat*)=2, and *freq*(*illness*)=*freq*(*sunny*)=1, respectively. The emotion intensity vector of each emotion word is *v*(*threat*)= <0.742*,* 0.604*,* 0*,* 0*,* 0*,* 0*,* 0*,* 0 *>*, *v*(*illness*)=<0*,* 0.609*,* 0.688*,* 0*,* 0*,* 0*,* 0*,* 0 *>*, and *v*(*sunny*)=*<*0*,* 0*,* 0*,* 0*,* 0.562*,* 0.5*,* 0 *>*. For each emotion word, we multiplied the emotion intensity vector with the frequency of the word and then computed the average emotion intensity for all emotion words. The average of all emotion word intensity for this user is *<*0.495*,* 0.605*,* 0.229*,* 0*,* 0*,* 0.187*,* 0.167*,* 0 *>*. We repeated the process for each user to obtain the user-level average emotion intensity to obtain affective features for all users.

Research shows that the occurrence and distribution of particular words within a document can represent its main content [48]. This suggests that the occurrence of a particular emotion word appearing in social media postings can indicate individuals’ emotional state, sentiment, and mood. Thus, we extracted emotion words from individual with and without depression to illustrate their respective emotional states and make a comparison between them. We divided the status updates into 2 datasets: one containing only the status updates of the individuals with depression and the other containing only the status updates of individuals without depression. We then computed the term frequencies of each emotion word based on these datasets from individuals with and without depression:



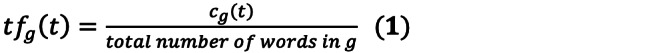



where *c_g_*(*t*) indicates the frequency of emotion words *t* in group *g* (*g*=*d* for the depressed group and *g*=*nd* for the nondepressed group).

Then, we find *df* (*t*), the ratio differences in relative term frequency for each emotion word *t* that appears in the datasets from individuals with and without depression as follows:

*df (t) = tf_d_ (t) – tf_nd_ (t)* **(2)**

We then sorted the ratio differences of all emotion words to find the most distinct emotion words across the depressed and nondepressed groups. We identify the top-k emotion words as distinct features of the depressed group and the bottom-k emotion words as distinct features of the nondepressed group, which help differentiate between the 2 groups.

**Figure 2:**
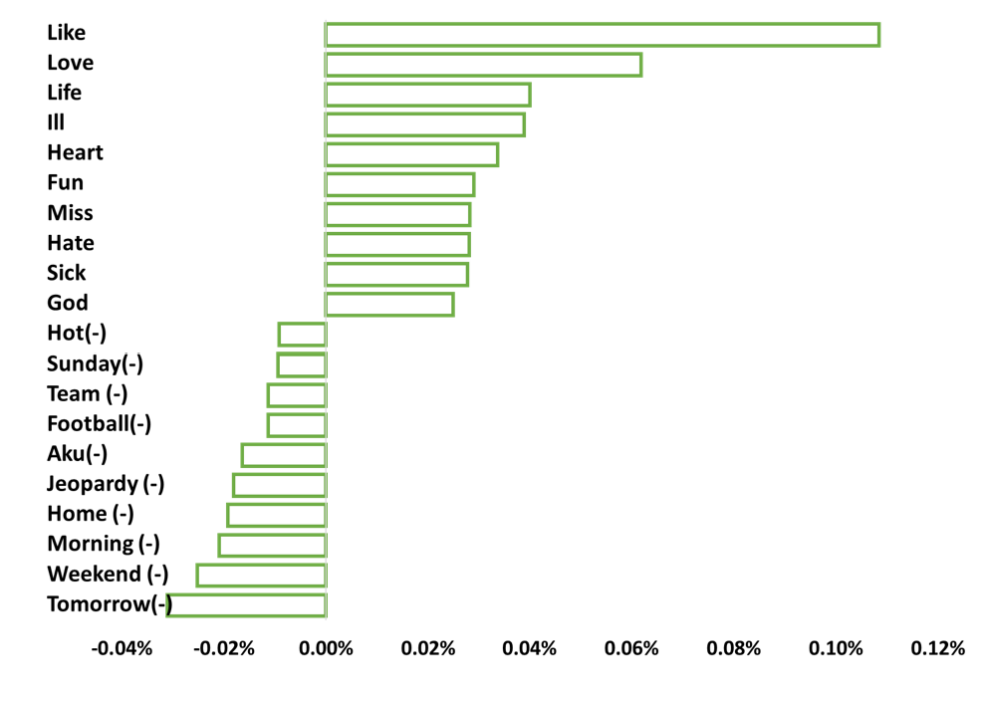
The top 10 distinct emotion words (positive) were associated with the group with depression, and the bottom 10 distinct emotion words (negative) were associated with the group without depression.

Figure 2 illustrates an example of the top 10 and bottom 10 emotions words. The emotion words such as “like,” “love,” “life,” “ill,” and “heart” have the most positive disparities in term frequencies between the depressed group and the nondepressed group. These words indicate the depressed group’s distinct emotion features. In contrast, “tomorrow,” “weekend,” “morning,” “home,” and “jeopardy” have the most negative differences, indicating the distinct emotion features of the nondepressed group. The distinct emotion features discovered through term frequency computation highlighted the contrasting emotional states between the depressed and nondepressed groups. These insights serve as invaluable resources for clinically intelligent decision-making, enhancing diagnostic accuracy and offering support tailored to the unique emotional needs of individuals, which, in turn, promotes more effective therapeutic outcomes and enhances overall well-being.

Once we identify the most distinct emotion features, we use term frequency and inverse document frequency (TFIDF) to indicate the importance of a word *t* to a document D (eg, users’ aggregated status updates) in a corpus [49]. Each user will have 1 feature vector with all emotion words based on the TFIDF (t, D) value:

*TFIDF (t, D) = TF (t, D) × IDF (t)* **(3)**

where TF (t, D) stands for the term frequency of word *t* in a specific document *D*. The higher TF *(t, D),* the more important the word *t* is for that document D*.* IDF (t) stands for inverse document frequency and shows how significant term *t* is in the entire collection of documents, with rarer words having higher IDF values.

### Discover Personality Features

This section outlines how we extracted personality features. Personality shows the patterns of individuals’ thoughts, feelings, and actions as well as their attitudes and reactions to external social environments. Numerous studies have shown a link between personality and depressive symptoms [28,29,50]. Therefore, many studies adopt personality for diagnosing depression [32,51].

Our study uses the IPIP questionnaire [42] to measure individuals’ personalities based on 5 dimensions outlined in the Big Five Factor Model [48]. This model is the most commonly accepted personality theory in various research studies and clinical practice, offering a comprehensive approach to characterizing an individual’s unique characteristics and patterns of behaviors. The model shows how people think, feel, and behave with 5 dimensions: extraversion, agreeableness, conscientiousness, neuroticism, and openness [52,53]. Preoţiuc-Pietro et al [54] used language-derived and user-level “big five” personality factor dimensions to predict the depression state of a user based on social media postings. The personality features show strong performance in differentiating between users with depression and without depression. Yang et al [55] also used personality indicators for depression detection, affirming their strong correlation with traits such as neuroticism, extraversion, and conscientiousness. The IPIP questionnaire includes 50 assessment questions designed to assess diverse individual differences. Participants indicate the extent of truthfulness regarding themselves on a scale from 1=disagree to 5=agree. Higher scores on a personality dimension indicate greater alignment with the respective trait. Multimedia Appendix 2 shows a detailed breakdown of each trait. Five numerical features are derived through responses to the IPIP questionnaire, each corresponding to a participant’s personality trait.

### Discover Contextual Features

This section shows how we extracted contextual features (contextual meaning of words derived from the words’ surroundings in a sentence), which is another critical component of user-generated content. To extract contextual-level language features to reflect different contexts and meanings of words in sentences, we considered the sequence of words in users’ social media postings to learn sequence-level semantics. Given that social media posts are often written in a general format, characterized by informal or everyday language, pretrained models—typically trained on general datasets—are particularly effective in capturing these patterns. Their strong contextual modeling capabilities have made pretrained language models well suited for analyzing such data, including the detection of different mental health conditions [51,56,57]. Thus, we take advantage of them to extract contextual-level language features from user generated content. Moreover, considering contextual information, we conducted cluster analysis to visualize specific words used by users with depression and without depression for further analysis by clinicians.

A state-of-the-art model such as Bidirectional Encoder Representations from Transformers (BERT) [58] is a pretrained language model designed to learn deep bidirectional representations of natural language from extensive unsupervised text corpora. BERT has the ability to capture information from both the preceding and following words, enabling it to grasp a more comprehensive understanding of the language context. BERT generates a representation for each word, called word embedding, which can be used for further ML tasks such as classification or clustering. However, to perform ML tasks such as classifying sentences, BERT cannot directly generate independent sentence embeddings (eg, representation for sentences). Thus, we adopted Sentence BERT (SBERT) [59], which effectively represents texts with >1 term. The SBERT model was fine-tuned and pretrained on large datasets [60,61]. Input sentences can be transformed into fixed-sized vectors (768 dimensions), serving as sentence representations. Moreover, sentences that are semantically similar will be close to each other; that is, if we use similarity measures such as Euclidean distance measure to compute the differences between 2 sentences, the more similar the 2 sentences are, the shorter the distance between them.

Multimedia Appendix 3 outlines the proposed procedure to discover contextual features. We first aggregated each user’s social media postings. Then, we applied SBERT for each user’s aggregated posting [59] to learn sentence embeddings based on each word’s contextual information in a sentence to obtain user-level contextual features.

User-level contextual features alone may not offer interpretable information for aiding clinicians in making informed decisions. Hence, based on the sentence embedding, we conducted a cluster analysis to examine the content discussed by different user groups. First, we split the sentence embeddings (eg, user-level contextual features) into 2 datasets (depressed and nondepressed). Next, we applied K-means clustering separately to the datasets of user groups with depression and without depression to cluster sentence embeddings from each group. This helps differentiate the contents or topics discussed by different user groups.

To accomplish this, K-means clustering is conducted across a range of cluster sizes, computing the corresponding silhouette scores—a well-known measure indicating how effectively the resulting clusters separate from each other. The best model is chosen based on the highest silhouette score, with the associated number of clusters representing the optimal cluster count for K-means clustering on the dataset.

For each user group, users belonging to the same cluster are grouped together to obtain the aggregated social media postings. Next, we identified the main topics that user groups with depression and without depression talk about within each cluster. This is achieved by extracting the top-*k* words from each cluster based on term frequency, indicating how often a term appears in a specific cluster and group. Subsequently, we analyzed the words and main topics frequently discussed by different user groups.

### Discover Social Interaction Influence Features

This section shows how we extracted social interaction influence features. Studies show that social interactions within social groups can directly or indirectly impact an individual’s mental state [33]. Online social interactions are considered as intentional social actions that individuals engage in within the context of participation [62]. These interactions include users expressing their feelings and emotions by posting status updates and comments to share conventions, norms, or languages with positive or negative moods in online social groups. Research indicates that social interaction can facilitate the transfer of negative moods between individuals [34]. Thus, individuals’ psychological patterns and the specific words and sentences they use in their online postings in different contexts may affect how friends or social connections in their online social groups react or express themselves on the internet. We conducted social interaction analysis to extract the social interaction influence features for each user in the social network and examine the impact from the psychological and contextual perspective of other users in social groups through social connections. Social interaction influences features that consider users’ psychological and contextual features and network structure from social connections.

Multimedia Appendix 4 shows procedures for obtaining social interaction influence features. For each user, we obtained feature vectors from psychological and contextual features described in the Discover Affective Features, Discover Personality Features, and Discover Contextual Features sections. For each type of feature *f* (eg, emotion, personality, or context features), we have 2 feature vectors, *FV^f^_U,d_*, and *FV^f^_U,nd_* for each user *U* where *g* ∈ {*d, nd*}. We then found the social connections of each pair of users via the user-friendship network that shows if one user is connected with the other. If user *U* is a friend of another user, *Y*, they are socially connected. Next, we examined the influence of users with depression and without depression on the target user *U*, respectively, based on the different features to obtain emotion, personality, and context influence features. We argue that the emotions, personality, and context information of users with depression and without depression on social media affect how other users express themselves through immediate and nonimmediate social connections and interactions. Therefore, if one user is more influenced by another, their features will be more similar. Moreover, the target user is more likely to be influenced by their close neighbors in a social network regarding distance. Thus, a more immediate neighbor influences the target user more than a faraway neighbor in the social network. Hence, we extract distance-weighted social interaction influence features from users with depression and without depression by considering both network structure (eg, distances between users) and different features (eg, emotions, personality, and contextual features) simultaneously to reflect the neighbors’ impact on the target user. We first run the shortest paths for the whole social network between each pair of users. For target user *U*, we obtained the distance measures of the target user and the neighbor user *Y* as *Distance* (*U, Y)*. Then, we assigned weight to each neighbor user *Y*.

Then, the influence of depressed and nondepressed neighbors on the target user was computed using the weight and feature vectors. Consequently, we derived 2 separate social interaction influence features for the target user to represent the degree of similarities between the target user and their depressed and nondepressed neighbors. The same procedure was applied to all social network users.

**Figure 3:**
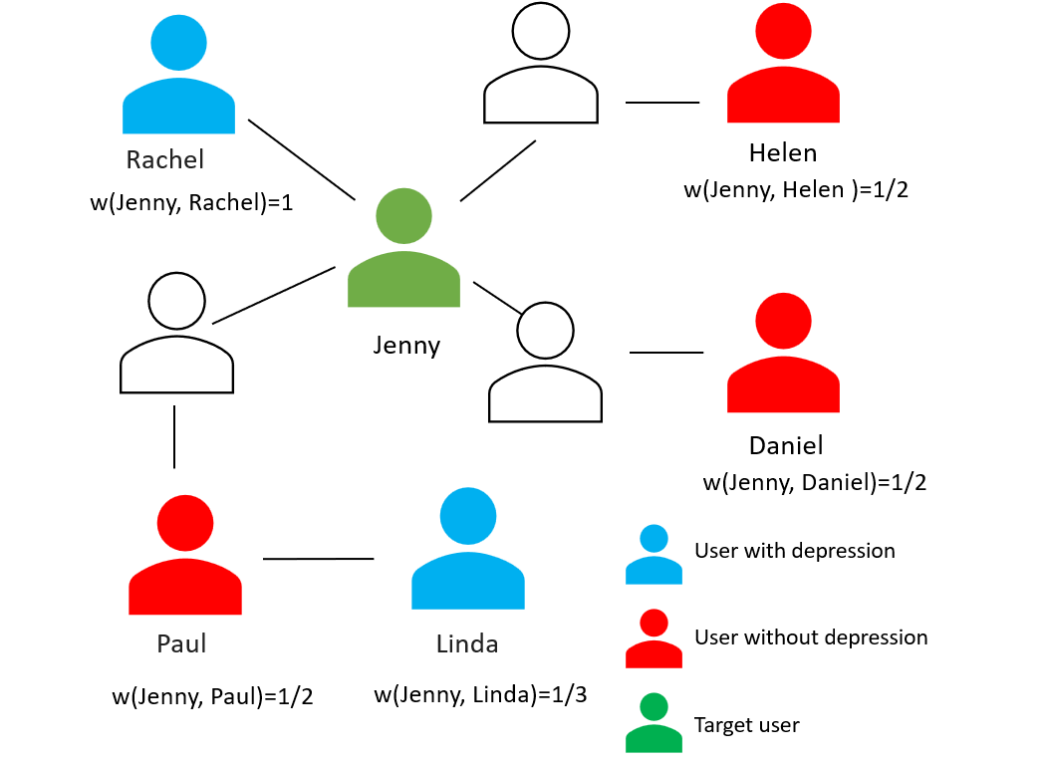
Partial user-friendship network of Jenny showing a direct connection to Rachel (immediate neighbor) and indirect connections to Paul, Helen, Daniel (second-level neighbors), and Linda (third-level neighbor) in her user-friendship network.



We illustrate how the analysis works with an example for a particular user, Jenny. Figure 3 shows a partial user-friendship network Jenny. She has a direct connection to Rachel (immediate neighbor) and indirect connections to Paul, Helen, Daniel (second-level neighbors), and Linda (third-level neighbor) in her user-friendship network. Because the shortest path between Jenny and Rachel (first-level neighbor) is 1, we consider their distance to be 1 (ie, *Distance* [*Jenny, Rachel*]=1). Because the shortest path between Jenny and her second-level neighbors (ie, Paul, Helen, and Daniel) is 2, we consider the distance as 2. Likewise, the distance between Jenny and her third-tier neighbor (ie, Linda) is defined as 3, and so forth. Because we argue that the target user is more likely to be influenced by their immediate neighbors in a social network, we can infer that the first-level neighbor, Rachel, has a greater potential to impact Jenny than the second-level neighbors, Paul, Helen, and Daniel. The second-level neighbors have a stronger influence over Jenny than the third-level neighbor, Linda. Consequently, we allocated a greater influence on the first-level neighbor than the second-level neighbor to reflect the higher impact first-level neighbors have on the target user. Similarly, we assigned a higher weight to second-level neighbors than third-level neighbors. For each neighbor *Y* of user *U*, we assigned scores based on the following equation:

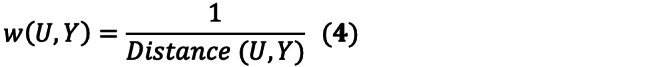



Thus, we assigned a weight of 1 to Rachel; a weight of 1/2 to Paul, Helen, and Daniel; and a weight of 1/3 to Linda to capture the decreasing influence with increasing distance between users.

In our analysis, we considered that the level of influence one user has on another is correlated with the similarity of features between the 2 users. Thus, we used Euclidean distance to assess the similarities of user *U* with their depressed and nondepressed neighbors separately. These similarities are determined by evaluating the depressed and nondepressed social media content of user *U* and their neighbor *Y* for each type of feature *f* (eg, emotions, personality, and contextual features). The similarity is calculated as the absolute difference between the feature vectors:



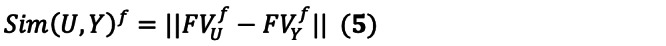



*Sim* (*U, Y*)*^f^* is calculated for all users in a social network with confirmed depression states. Then, we combined similarity measures with weights to obtain the social interaction score:



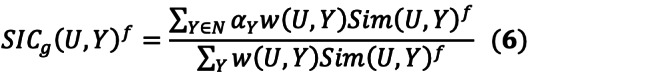



where *α_Y_* is an indicator and *α_Y_*=1 when the neighbor belongs to user group *g*. The higher the values of *SIC_g_*(*U, Y*), the more impact the specific neighbor user group (depressed or nondepressed) has on the target user in the social network. Multimedia Appendix 5 shows the pseudocode of the proposed social interaction analysis.

### Ethical Considerations

The data used in this study were originally collected by researchers at Johns Hopkins University and received approval from their institutional review board. Permission to use the data solely for research purposes was obtained under a confidentiality agreement. This paper details the privacy-preserving procedures used to maintain participant anonymity in the Data Cleaning and Preparation section. Because this study only reanalyzed secondary, anonymized data, additional ethics approval for this study was not required. The original data host has obtained informed consent from all participants who voluntarily donated their data for research purposes, permitting reuse for academic research. The dataset used for this secondary analysis consisted of fully anonymized data and was restricted to noncommercial academic research purposes. This secondary analysis did not involve any direct participant interaction or additional compensation. There is no identification of individual participants or users in any images or supplementary material included in the manuscript. The dataset was deidentified, ensuring no identifiable information was presented.

## Results

### Overview

In this section, we follow the feature discovery procedure discussed in the Methods section and analyze the proposed features. We then conduct experiments to evaluate the proposed framework based on the datasets [40], which include Facebook users’ status updates, network connections, users’ associated depression scores measured by CES-D questionnaires, and personality measured by the IPIP personality questionnaire. We demonstrate how we extract psychological and contextual features as well as the resultant social interaction influence features. We explore the understandability of the developed features in explaining and understanding users’ sentiments, moods, emotional behaviors, psychological states, and influence from social interactions in social networks. Our framework can be extended to include any social media and social platforms to enhance the capability of mental health professionals and clinicians to make explainable and timely diagnoses, referrals, interventions, and treatments for their potential patients.

### Analysis of Affective Features

**Figure 4:**
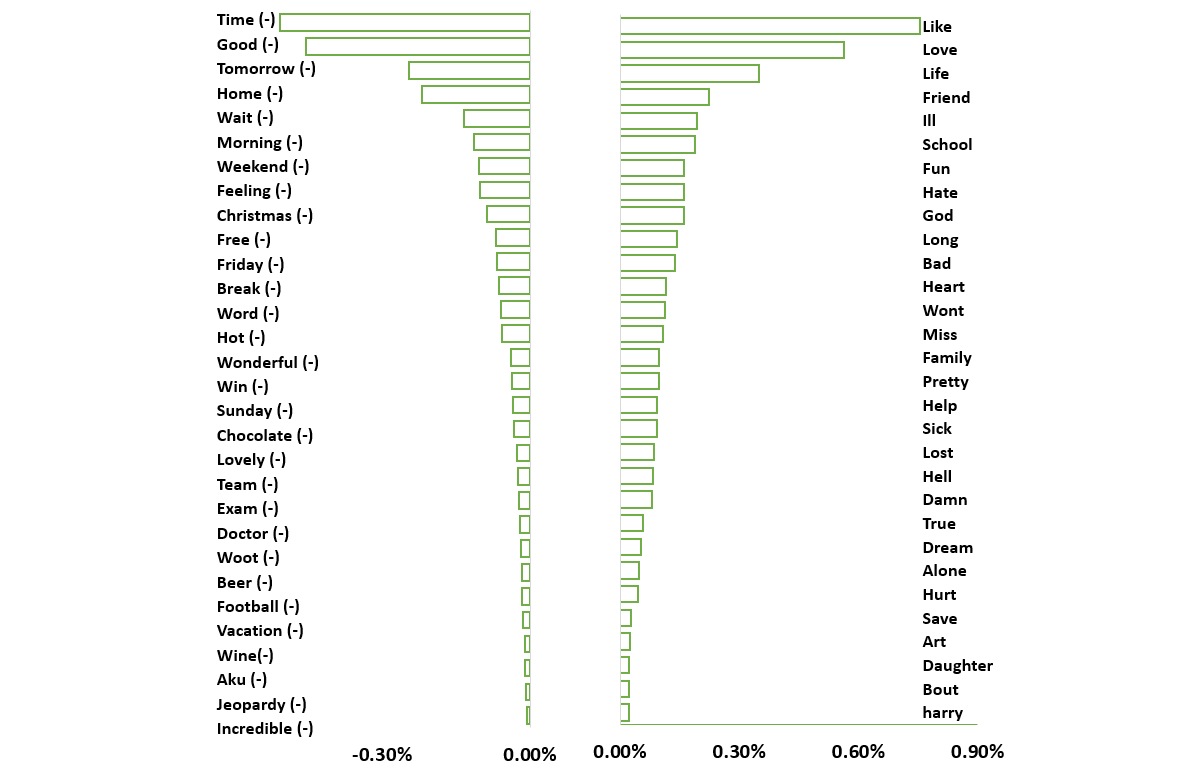
Emotion frequency distributions of the user groups with depression and without depression. The top 30 distinct emotion words (positive) are associated with the depressed group, and the top 30 distinct emotion words (negative) are associated with the group without depression.

Given the procedure outlined in the Discover Affective Features section, we first obtained user-level emotion intensity by averaging the emotion intensities of the 8 emotion dimensions in users’ social media postings. We further explored emotions, sentiments, and moods across different user groups by examining the top 30 and bottom 30 emotion words as distinct features, which were extracted from the depressed and nondepressed groups, respectively. Figure 4 illustrates the frequency distributions for each user group. Words with higher frequency values were more distinct emotion terms in each user group. The user group with depression frequently used emotion words with negative sentiments, such as “sick,” “hurt,” “bad,” “alone,” “ill,” “lost,” and “hate,” and some swear words, which align with those reported in previous studies [63]. In contrast, users without depression expressed positive sentiments, including “good,” “wonderful,” “incredible,” and “lovely.” Words such as “family,” “friends,” and “daughter” were frequently mentioned in status updates by individuals in the user group with depression, indicating specific individuals or individuals that are related to them. In contrast, events such as “exams” and “vacation”; specific food, drinks, or items related to events including “beer,” “wine,” “football,” “chocolate,” and “hot”; and outcomes of events such as “win,” “break,” and “jeopardy” were discussed by the user group without depression.

From Figure 4, we observe that the user group without depression is more likely to use emotion words associated with time, such as “time,” “tomorrow,” “morning,” “weekend,” “friday,” “christmas,” and “sunday.” In contrast, the user group with depression expresses feelings by using words such as “like,” “love,” and “fun.” They also care about more internal things such as “god,” “life,” “hear,” and “dreams.”

We calculated TFIDF (t, D) for each emotion word *t* in each user’s social media postings *D* to generate the feature vectors based on the most distinct depressed and nondepressed words as inputs for classifying the users with and without depression.

We conducted further analysis to examine the emotion intensities for each user group separately. To achieve this, we calculated the average intensity of each word in the emotion dimensions for the top 30 words in the depressed user group and the bottom 30 words in the nondepressed user group. The set of distinct emotion words used by user group *g* is *{T_g1_, T_g2_, ..., T_g30_}*, where *g* ∈ {*d, nd*}. For each emotion word *t_g_* ∈ *{T_g1_, T_g2_, ..., T_g30_}* in group *g*, we denoted the total count of word *t_g_*’s appearance in all users’ social media postings in group *g* by *TC_tg_* and the total number of words for all users as TC*_U_*. Then, the average intensity of in group *g* is computed as: Sum of:

(TC_tg_ * V(t_g_))/TC_U_ **(7)**

**Figure 5:**
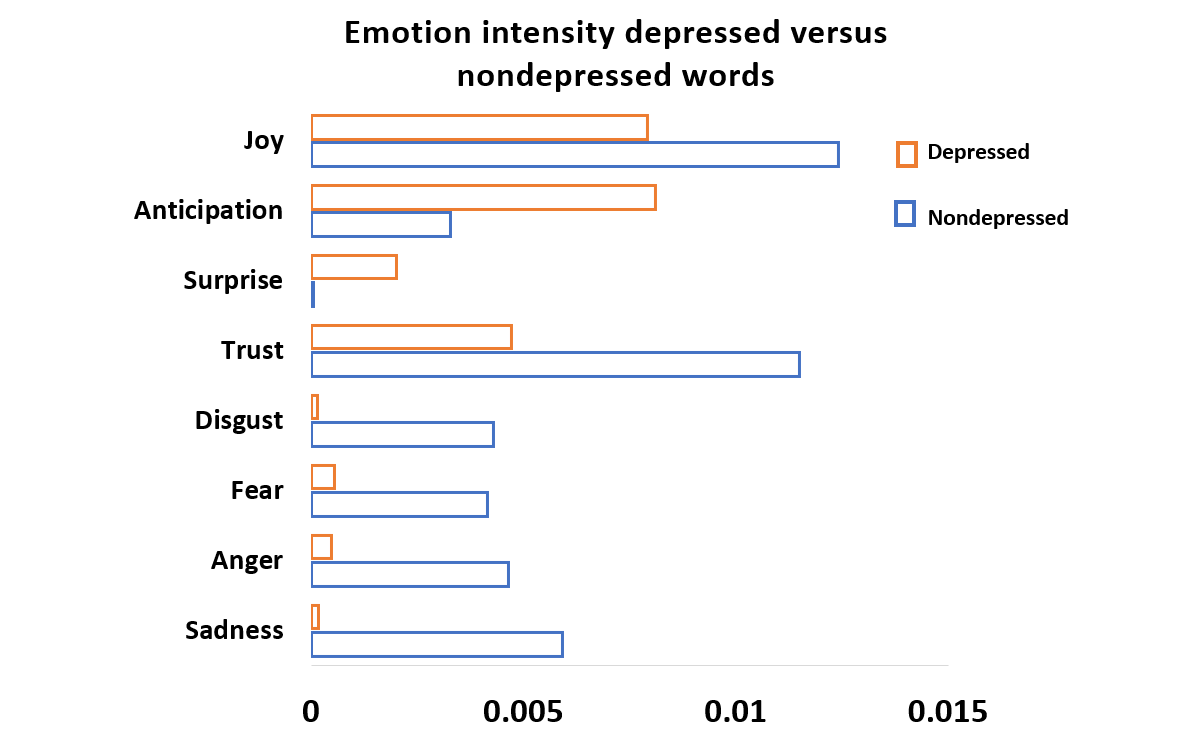
Comparison of average emotion intensities of the most distinct emotions of user groups with depression and without depression.

We compared the average intensities of the most distinct emotions of these 2 user groups, as shown in Figure 5. Distinct depressed emotion words have high intensities in all emotion categories, suggesting that the user group with depression tends to have more emotional behavioral changes than the user group without depression. The user group with depression shows significant intensities in sadness, anger, fear, disgust, and trust compared to the user group without depression, suggesting that negative emotions are frequently expressed through social media postings by the user group with depression. The user group with depression also shows a higher intensity of joy than the user group without depression. This suggests that users with depression may try to use words related to joy to cheer themselves up, or they are seeking joy.

### Analysis of Personality Features

As discussed in the Discover Personality Features section, we used the IPIP questionnaire to assess individuals on 5 dimensions of personality according to the Big Five Factor Model. For each user, we obtained a score for each of the 5 personality dimensions to form a feature vector for the personality feature of that user. Table 1 provides an example illustrating how we obtain the feature vector for each user. This personality feature vector comprises 5 values, each representing 1 of the 5 personality dimensions. The score for each dimension ranges from 1 to 5. For example, as shown in Table 1, the first user, *U*_1_, scores 4 for openness, 2.25 for conscientiousness, 2.2 for extraversion, 3.6 for agreeableness, and 2.8 for neuroticism. Thus, *U*_1_’s feature vector for personality is *<*4*,* 2.25*,* 2.2*,* 3.6*,* 2.8 *>*. Similarly, we obtained the feature vector for *U*_2_ as *<*3.8*,* 3.75*,* 3.4*,* 4.4*,* 2.15*>* and for *U*_3_ as *<*3.55*,* 3.2*,* 3.2*,* 3.35*,* 2.9*>*, and so on. The feature vector for personality will be used to run experiments for depression detection.

**Table 1:** Examples of personality feature vectors for 3 users across 5 personality traits.

User	The feature vector <openness, conscientiousness, extraversion, agreeableness, neuroticism> is a numerical representation of personality traits based on the Big Five model used to describe and analyze individual characteristics in a structured format.
U_1_^a^	*<*4*,* 2.25*,* 2.2*,* 3.6*,* 2.8*>*
U_2_	*<*3.8*,* 3.75*,* 3.4*,* 4.4*,* 2.15*>*
U_3_	*<*3.55*,* 3.2*,* 3.2*,* 3.35*,* 2.9*>*

^a^U_1_ represents first user, U2 represent second user, and so on.

### Analysis of Contextual Features

As outlined in the Discover Contextual Features section, we first used SBERT [59] to learn sentence embeddings based on each word’s contextual information in a sentence for each user’s social media posting to obtain user-level contextual features. The sentences users discuss in their social media postings are transformed into fixed-sized vectors (768 dimensions), which are the representation of the sentences. We conducted cluster analysis based on the SBERT feature vectors to obtain more valuable insights to support clinical decisions. We conducted K-means clustering separately for the user groups with and without depression to analyze different contents or topics different user groups discuss on social media. We found the optimal number of clusters for each user group based on the “silhouette” method. We chose 2 and 4 to be the optimal number of clusters for the user groups with and without depression, respectively, by experimenting with silhouette scores with regard to a range of clusters (ie, we choose the number of clusters with the highest silhouette score as the optimal number).

We then grouped users belonging to the same cluster together and obtained the aggregated social media postings for each cluster. Because the nondepressed group had fewer clusters than the depressed group, we obtained the top 20 words from each depressed cluster based on the term frequency and the top 40 words for each nondepressed cluster to have an equal number of top words from each user group for comparison.

The top words discussed in each cluster for each user group are shown in Multimedia Appendices 6 and 7.

On the basis of the top words, we conducted further analysis. Swear words and negative sentiments, such as “ass,” “f**king,” and “tear,” were unique to the user group with depression, as shown in Textbox 1. This group also uses words related to family members, such as “dad” and “daughter,” and spiritual words, such as “pray,” “bless,” and “trust.” This may suggest that the user group with depression tends to seek comfort from family or the spiritual world. They also talk about school-related terms such as “teacher” and “college.” This might suggest that the user group with depression has depressed symptoms due to pressure from school. The non-depressed group typically uses fewer negative sentiment words. The user group without depression uniquely mentioned words related to being forward-looking or future, such as “future,” “forward,” “chance,” and “step”; optimistic actions, such as “pass,” “win,” and “worth”; or positive words, such as “power” and “perfect.” They also used words suggesting the beginning of something, such as “awake” and “begin.”

**Comparison of unique words used by users with and without depression.**

**Unique words used by users with depression**

- “Awake”, “begin”, “burn”, “chance”, “comment”, “cut”, “exam”, “Facebook”, “fear”, “final”, “forward”, “future”, “human”, “pass”, “paste”, “perfect”, “point”, “power”, “problem”, “self”, “side”, “step”, “thought”, “truth”, “weather”, “win”, and “worth”.

**Unique words used by users without depression**

- “Ass”, “band”, “beat”, “bless”, “dad”, “daughter”, “chocolate”, “college”, “easy”, “fly”, “fxxking”, “kind”, “omg”, “pray”, “repost”, “seriously”, “tear”, “teacher”, “text”, and “trust”.

### Analysis of Social Interaction Influence Features

Next, we used the proposed psychological and contextual features, along with the social network structure derived from social connections, to extract social interaction influence features. We created social interaction influence features for each user and each type of proposed feature to show the extent of influence exerted by user groups with and without depression on the target user. As shown in Table 2, the example illustrates the affective, personality, and contextual influence of neighbors with and without depression, respectively, on 4 users, *U*_1_ (user 1), *U*_2_ (user 2), *U*_3_ (user 3), and *U*_4_.(user 4) We first extracted each user’s affective, personality, and contextual features. For each user, *U_i_*, and each feature, *f*, we obtained feature vectors *FV^f^_U,d_* and *FV^f^_U,nd_* that represent the features from user *U_i_* ’s social connections with and without depression, respectively.

**Table 2:** Examples of affective, personality, and contextual influences on four users, stemming from interactions with neighbors with depression and without depression, respectively. Each value represents the proportion or impact (as a fraction of 1) of the specific influence type (affective, personality, contextual) on the given user from neighbors with or without depression.

User	Feature type							
	Affective			Personality			Contextual	
	Depressed	Nondepressed	Depressed		Nondepressed	Depressed		Nondepressed
*U_1_^a^*	0.736	0.264	0.677		0.323	0.668		0.332
*U*_2_	0.566	0.434	0.514		0.486	0.396		0.604
*U*_3_	0.282	0.718	0.405		0.595	0.464		0.536
*U*_4_	0.206	0.794	0.191		0.809	0.265		0.735

^a^U_1_ represent first user, U2 represent second user, and so on.

These feature vectors are inputs for extracting the social interaction influence features. In the case of affective features, the scores obtained from the users’ group with depression are higher than those from the users’ group without depression for *U*_1_ and *U*_2_. This suggests a stronger social interaction influence from the user with depression compared to the user without depression for *U*_1_ and *U*_2_. As a result, these 2 users exhibited emotions that are closer to those of their friends with depression and further from those of their friends without depression. In contrast, *U*_3_ and *U*_4_ exhibited greater similarity to their friends without depression compared to their friends with depression, suggesting they experienced a stronger influence from the friends without depression. Thus, *SIC_d_(U,Y)* affective is larger than *SIC_nd_(U,Y)* affective for user *U*_1_ and *U*_2_ for emotion features. Likewise, for personality and contextual features, higher scores suggest a higher influence. By comparing the scores between the user groups with depression and without depression, a higher score for a feature in 1 group suggests that the users are more aligned with that particular user group regarding that feature. This observation suggests that within the social networks, the user groups with depression and without depression may have different effects on their friends. Users who are more influenced by the user group without depression tend to display a higher degree of resemblance in affective, personality, or contextual information with the user group without depression, and vice versa.

### Experiment Results: Depression Prediction Using ML Methods

The proposed psychological and contextual analysis NLP framework has the potential to enhance intelligent decision-making in the mental health domain. Our framework can enhance the reliability and knowledge base of decision support systems for health care providers, facilitating more accurate diagnoses and timely referrals and interventions, while also simplifying treatment plans. We found that incorporating affective, contextual, and personality features outperformed traditional baselines (improving performance compared to traditional baselines with an average improvement of 6% in accuracy and 10% in *F*_1_-score) and achieved performance comparable to state-of-the-art baselines. Furthermore, the addition of social interaction influence enhanced performance across all metrics.

We conducted experiments using ML methods with the proposed features for depression detection to demonstrate the performance of our framework. All ML implementations were conducted using Python’s *scikit-learn* library. The experiments were conducted on a ThinkPad (X1 Yoga Gen 8 Intel) with an Intel Core i7 CPU, 16 GB of RAM, running Windows 10. We used 5-fold cross-validation for model evaluation. Four ML methods were used: (1) logistic regression, (2) neural network, (3) k-nearest neighbors (KNN), and (4) random forest. We used an 80/20 split for training and testing the models. Specifically, we reported the accuracy of depression detection as part of our evaluation:



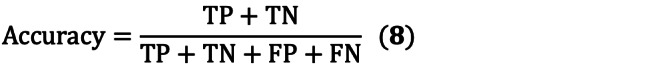



where TP=true positives, TN=true negatives, FP=false positives, and FN=false negatives.

In addition, we reported recall and precision scores. The recall represents the ratio of correctly predicted users with depression among all actual users with depression, while precision measures the proportion of users with depression correctly identified as depressed relative to the total number of users predicted as depressed, that is:



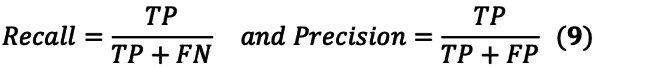



We also reported *F*_1_-score, which is the combination of precision and recall:



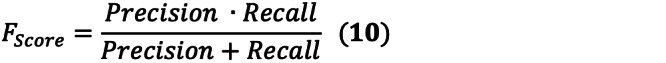



To assess the predictive capability of our proposed framework, we leveraged psychological (eg, affective and personality), contextual, and social interaction influence features to detect depression. We used the Linguistic Inquiry and Word Count (LIWC) [64] feature as the baseline, which is easily replicable to provide a comparative benchmark with the same data. The LIWC feature is widely used in depression detection because it offers reliable, unbiased, and effective measures [65-68].

**Figure 6:**
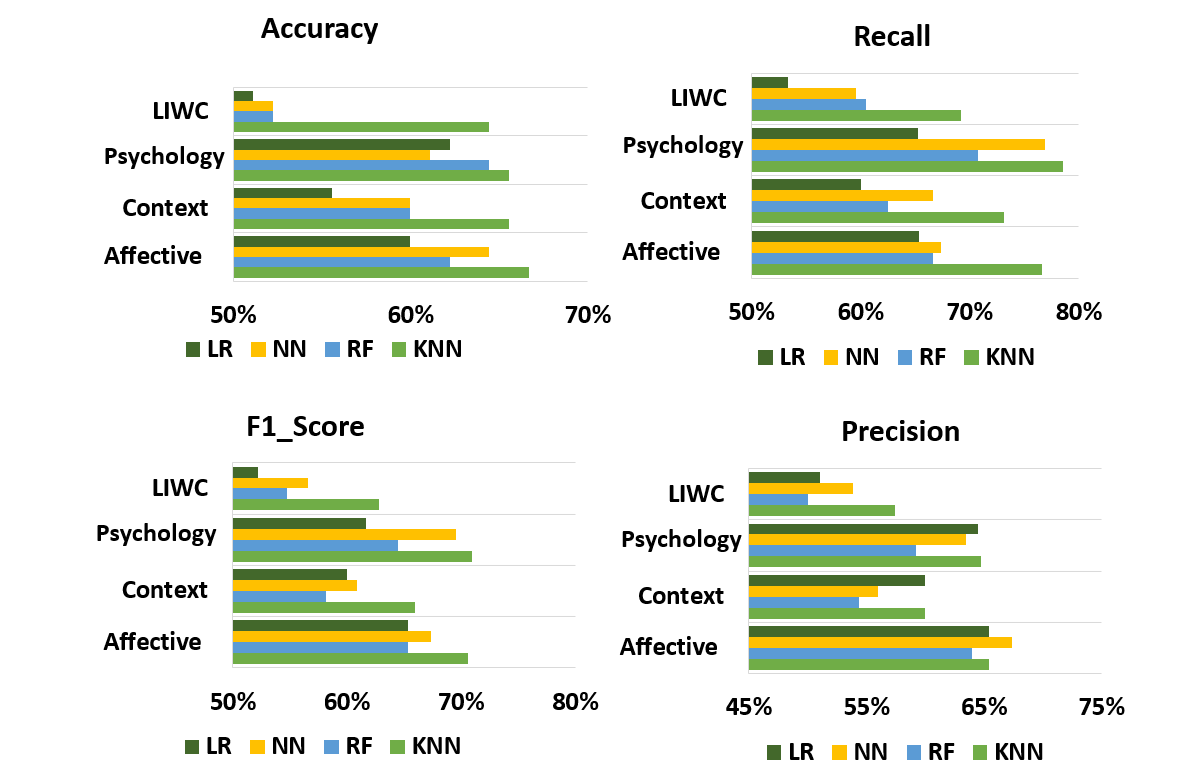
Performance comparison of different machine learning methods across 4 metrics using Linguistic Inquiry and Word Count (LIWC), personality (psychology), contextual, and affective features. KNN: k-nearest neighbors; LR: logistic regression; NN: neural network; RF: random forest.

We first conducted experiments using psychological (eg, affective and personality) and contextual features. The performance of various ML methods for different sets of features is illustrated in Figure 6. KNN showed the highest accuracy among all other ML models, with 69% for the LIWC feature, 76.6% for the affective features, 73% for contextual features, and 79% for the personality features. For precision, although neural network showed a higher performance of 67% compared to 66% under KNN for the affective features, KNN showed better performance compared to other ML methods for all other features. KNN also indicated the highest recall and *F*_1_-score compared to other ML methods among all feature sets. Overall, KNN performed the best among all ML methods used in this study. Therefore, we conducted an additional set of experiments with different feature sets (F1-F9 correspond to different feature sets) listed in Table 3, using KNN, to assess the predictive power of depression detection based on the proposed features for each feature set.

**Table 3:** Different feature sets using KNNa to evaluate the predictive power of depression detection.

Feature short name	Feature variables
Baseline1	LIWC^b^ features
Baseline2	FastText
Baseline3	CNN^c^
F1	Affective features
F2	Contextual features
F3	Personality features
F4	Affective features, contextual features, and personality features
F5	Affective influence features, contextual influence features, and personality influence features
F6	Affective features and affective influence features
F7	Contextual features and contextual influence features
F8	Personality features and personality influence features
F9	Affective features, contextual features, and personality features and affective influence features, contextual influence features, and personality influence features

^a^KNN: k-nearest neighbors.

^b^LIWC: Linguistic Inquiry and Word Count.

^c^CNN: convolutional neural network. F1-F9 correspond to different feature sets listed in Table 3, F1 is feature set 1 which is Affective Features as listed in Table 3, F2 is feature set 2 which is listed as Contextual Features in Table 3 and so on.

We treated the LIWC feature as baseline 1 and compared it against the proposed affective, contextual, personality, and corresponding social interaction influence features (eg, affective influence, contextual influence, and personality influence features) using KNN. We also compared our model with state-of-the-art approaches that used pretrained models for text processing combined with ML techniques for detection, such as FastText with XGBoost (baseline 2, as used in the study by Marriwala and Chaudhary [69]) and deep learning architectures, including convolutional neural networks for text processing and detection (baseline 3, as described in the study by Ghosal and Jain [39]).

**Figure 7:**
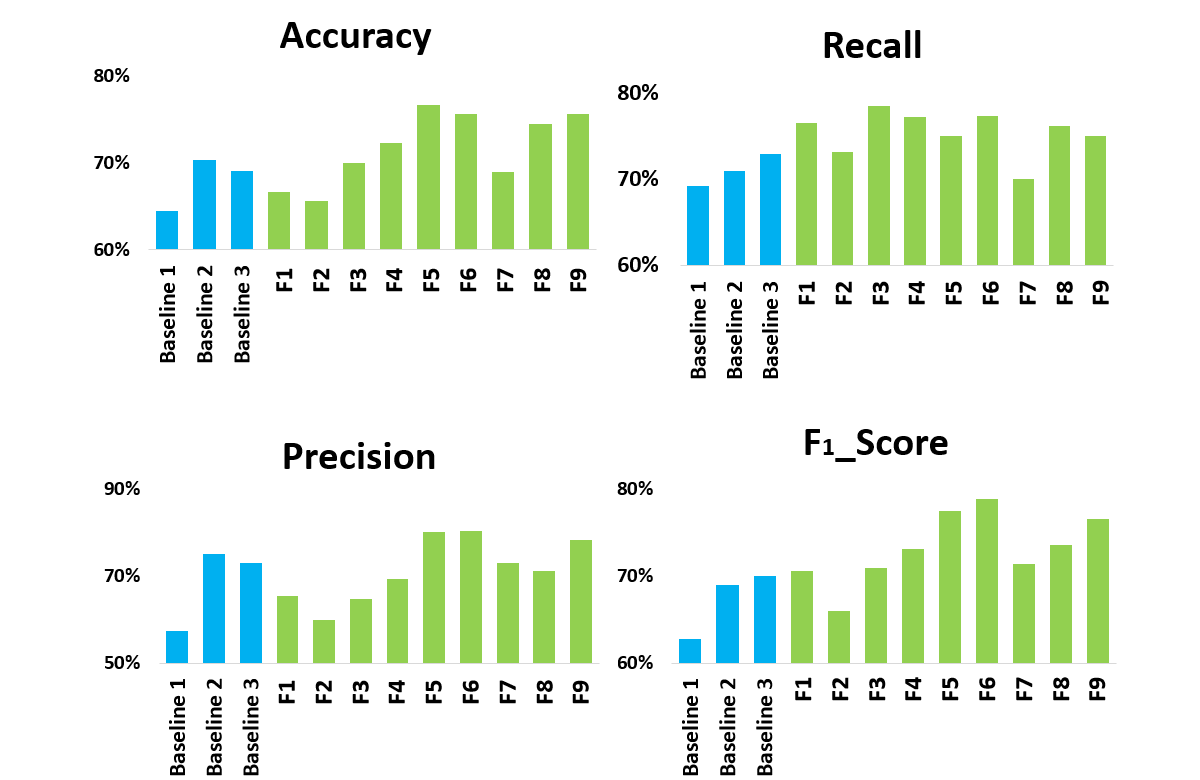
Performance comparison across different feature sets and baselines.

As shown in Figure 7, the feature sets incorporating the proposed features significantly improved the detection capability compared to the LIWC feature across all performance measures. Our results are comparable to those achieved by baseline 2 (FastText with XGBoost) and baseline 3 (convolutional neural networks); however, the combination of all affective, personality, and contextual features, along with their influence features, yields even stronger performance. The inclusion of emotional and personality-driven factors, combined with social interaction features within our framework, offers a unique advantage. This approach delivers richer insights and a higher interpretive value compared to previous models, while maintaining high interpretability—a crucial aspect for mental health applications.

Among the proposed features, the model with affective features (F1) and the model with personality features (F3) performed better than the model with contextual features (F2). The model with affective features, personality features, and contextual features (F4) had the best performance in all measures except recall compared to F1, F2, and F3, while F3 showed better performance in recall compared to F4. The relatively strong performance of F2 showed the model’s ability to effectively distinguish users with depression, suggesting its capacity to identify depression-specific language patterns. When considering the social interaction influence features for each feature type, the model with affective influence, personality influence, and contextual influence features (F5) showed higher performance compared to F4 with respect to accuracy, precision, and *F*_1_-score. F3 and F4 showed better recall compared to F5. Compared to affective features (F1), the combination of affective features and the corresponding social interaction influence of affective features—affective influence features (F6)—performed better across all performance metrics. The same pattern applies for F7 with the combination of contextual features and the corresponding contextual influence features and for F8 with the combination of personality features and the corresponding personality influence features; they both performed better compared to the model with contextual features (F2) and personality features (F3) alone across all performance metrics. The model with all proposed features, including affective, personality, contextual, and the corresponding social interaction influence features (F9), showed high performance but not the highest among all models; this implies that simply including all features may not necessarily enhance performance. All proposed features outperformed baselines; notably, F5 and F6 demonstrated the strongest overall performance.

To examine how each feature impacts model performance, we performed permutation importance. This approach provides insights into how each feature affects the model’s accuracy by measuring the change in performance when individual features are randomly shuffled [70]. Multimedia Appendix 8 shows the top 10 influential features, highlighting those most impactful for depression detection. The results indicate that personality features such as neuroticism, extraversion, and agreeableness are highly significant, followed by social interaction influence based on emotion, personality, and contextual measures. Finally, the affective feature, anticipation score, is the most influential among all emotions. F1-F9 represent short name of 9 feature sets we used in the experiments

## Discussion

### Principal Findings

We contribute by proposing a methodological framework using NLP and ML techniques to extract features and patterns, thereby comprehensively understanding depression-related symptoms from psychological and contextual perspectives. Building on the foundations of social influence theory, which points out that the way individuals behave, think, or act is influenced by their social environment, we extracted social interaction influence features based on social media postings. These features considered both psychological and contextual features (eg, users’ emotions, psychological states, and context of communication) from users’ social media postings and the social network structure of social media. They reflected the influence that a user’s social connections may have on their mental well-being. The combination of all affective, personality, and contextual features, along with their influence features, yields strong performance compared to baselines. F5 (which represent affective influence features, contextual influence features, and personality influence features in Table 3) and F6 (affective features and affective influence features) show particularly strong performances. Our study, designed to complement clinical diagnoses, delivers results that are sufficiently robust to support timely interventions and practical applications in mental health monitoring. By achieving comparable results to state-of-the-art methods, our approach demonstrates strong performance while adding value through richer insights and high interpretability. These patterns bridge the gap between model accuracy and interpretability, providing more actionable and inseparable insights that can aid clinicians in better understanding users’ mentality and supporting their decision-making processes. This framework has the potential to expedite the diagnostic process and provide timely referral services to individuals at high risk, facilitating early intervention or treatment before more severe mental health conditions develop. With an improved understanding of users’ mental states reflected in the psychological and contextual aspects, clinicians can more easily detect significant changes in users’ mental well-being. This allows clinicians to provide well-supported guidance during early intervention and facilitate follow-ups in treatment plans.

Research in psychology has verified the theoretical connections between patients’ mental health conditions and certain linguistic features. Various studies establish that individuals experiencing certain mental illnesses exhibit specific verbal behavior. Therefore, there is an increasing trend toward using rich sources of text data and information from social media to examine users’ sentiments and behaviors in mental health studies [16,17]. Studies show that frequent negative affect expression has higher connections with depressive states, while positive affect tends to have a lower connection to depression [23-25]. Thus, emotions play an essential role in diagnosing mental illnesses such as depression [11]. Various studies have characterized emotions from language use of users disclosing their mental illnesses on social media to detect depression [68,71,72]. Personality is another important aspect of analyzing depression. Personality has been used to distinguish social media users’ activities, posting content, and language use habits [26,27,73]. Several studies show a link between personality types and depression, and personality features can be used in depression diagnosis and treatments [28,50,55].

In addition to psychological factors, pretrained language models have shown strong contextual modeling ability; they are used in understanding texts in various applications [58]. For example, Haque et al [74] leveraged pretrained language models to detect early signs of suicidal thoughts, improve the diagnosis process, and provide people with appropriate treatment on time. Jiang et al [75] used deep contextualized pretrained word representation to build strong predictive models to detect different mental health problems from social media. Murarka et al [37] built a multiclass model using the pretrained language model to classify 5 prominent types of mental illnesses by analyzing unstructured user data on Reddit. Social media plays a critical role in shaping attitudes, beliefs, and behaviors through interactions [76,77]. Social influence theory suggests that individuals’ mental states and social interactions affect their language and behaviors on social media [32,78]. While previous research has focused on linguistic, emotional, and personality factors, it often overlooks the impact of social interactions [37,68]. Our study aims to bridge this gap by analyzing social influence and interactions alongside psychological and contextual factors. This comprehensive framework can aid in improving mental health diagnosis and treatment plans.

Our study has several practical implications. By providing mental health professionals with a deeper and more comprehensive insight into a user’s mental state, our framework can help accelerate the depression diagnosis process. It also enables timely referral services to individuals who may be unaware of their mental states or have limited access to mental health resources. Moreover, it is beneficial to alert health care management to offer early intervention or treatment to individuals at risk of developing more severe mental health conditions. Furthermore, by gaining a deeper insight into users’ behaviors reflected in different aspects, mental health professionals can identify the significant changes in users’ emotional, psychological, and mental health states to provide more justified recommendations during the early intervention and facilitate easier follow-ups in treatment plans.

Other domains that analyze social interactions and language use can also adopt our framework. In the general health domain, our framework offers valuable assistance in discovering positive and negative opinions by studying user-generated content from health-based social platforms. These platforms serve as spaces where users, including patients, physicians, and caregivers, can pose questions and seek advice from other users. By analyzing emotions, personality, and contextual information and the influence of social interaction, medical professionals and caregivers can effectively monitor different health conditions among patients and individuals at risk. This allows them to provide timely advice, referral services, or interventions, ultimately enhancing health care quality in these online communities. In the finance domain, for example, StockTwits is an online platform where investors exchange ideas, opinions, and sentiments regarding financial markets [79]. Our framework can be used to examine how emotional shifts, personality differences, and contextual information within sentences influence investors’ decision-making processes. It also enables the examination of how social interactions among different investors may influence their investment decisions.

### Limitations

While analyzing users’ postings on social media enables the identification of mental states through means unavailable offline, our research does not consider the socioeconomic status, idiosyncratic behaviors “behind the scenes,” or dynamic language data. For future studies, complementary sources of behavioral data and dynamic data, such as activity logs or search history, could be integrated with health records, such as records of prescriptions or health care claims data, to offer a more comprehensive understanding of the mental health status of an individual. Moreover, the expression of individuals with depression may vary across different intensity levels of their depression. As a result, investigating how language use differs and evolves across different stages of depression could be a promising future direction, potentially leading to more personalized treatment plans. In addition, we plan to incorporate domain-specific lexicons and more specialized datasets to further enhance the model’s accuracy in capturing nuanced depressive language. We also aim to fine-tune SBERT embeddings using mental health–specific corpora, which could improve performance in detecting depression-related language. Furthermore, after comprehensively understanding the patterns and trends of users with depression, a database system can be developed to store significant behaviors or language use changes. Updated classifiers could be trained using real-time data from online platforms or other sources. The system could automatically detect users who are at risk of depression even when they are unaware of their mental conditions and recommend suitable treatments based on information extracted from previous instances. We also plan to incorporate more recent and real-world datasets with similar features, extending the analysis beyond mental health contexts to various domains of social network analysis. This broader approach will facilitate a more comprehensive exploration across diverse scenarios, enhancing the framework’s relevance and adaptability.

### Conclusions

Our study illustrates a psychological and contextual analysis NLP framework that uses affective, personality, and contextual information. We further explore the influence of social interaction shaped by these elements to monitor depression behaviors on social media using NLP and ML. The proposed framework highlights the potential of using emotion intensities, personality measurement, pretrained language models, and social interactions among users on social media as features or patterns for predicting depression in individuals. Our research shows the potential of developing a better foundation for predicting the risk of depression and offering referrals, early intervention, and treatment plans via the analysis of social media data. It presents promising potential as an intelligent decision-making tool integrating information from multiple channels, such as social networks.

## Data Availability

The datasets generated and analyzed during this study are available from the corresponding author on reasonable request.

## Appendix

Multimedia Appendix 1 The process of extracting emotion intensity features and emotion word features to represent the affective features. We extracted emotion words from social media posts of users with and without depression and calculated the ratio differences in relative term frequency to identify distinct emotion words. We also combined the intensity levels across 8 emotion dimensions with emotion word frequency, computing the average intensity to form an emotion intensity feature.

Multimedia Appendix 2 The Big Five Factor Model—personality traits with descriptions, showing the behavioral tendencies associated with low and high scores for each trait.

Multimedia Appendix 3 Discovery process of contextual features. We aggregated each user’s social media postings and applied Sentence Bidirectional Encoder Representations (SBERT) from Transformers to generate sentence embeddings, capturing the contextual information of each word within a sentence. These embeddings serve as user-level contextual features. Then, we performed cluster analysis using K-means clustering, applied separately to the sentence embeddings of user groups with depression and without depression to analyze the content discussed by different user groups.

Multimedia Appendix 4 Discovery process of social interaction influence features. For each user, we obtained feature vectors from the psychological and contextual features identified earlier. We then analyzed the influence of users with depression and without depression on the target user separately, based on the emotion, personality, and context influence features.

Multimedia Appendix 5 Algorithm for discovering social interaction influence features.

Multimedia Appendix 6 The most frequently discussed words across the four clusters for the user group with depression.

Multimedia Appendix 7 The most frequently discussed words across the two clusters for the user group without depression.

Multimedia Appendix 8 The top 10 influential features, highlighting the most impactful features for depression detection.

## Abbreviations

BERT: Bidirectional Encoder Representations from Transformers
CES-D: Center for Epidemiologic Studies Depression
IPIP: International Personality Item Pool
KNN: k-nearest neighbors
LIWC: Linguistic Inquiry and Word Count
ML: machine learning
NLP: natural language processing
SBERT: Sentence Bidirectional Encoder Representations from Transformers
TFIDF: term frequency and inverse document frequency

## References

[1] Williams JW, First M, McNutt JG (2013). Diagnostic and statistical manual of mental disorders. Encyclopedia of Social Work.

[2] Zhang T, Schoene AM, Ji S, Ananiadou S (2022). Natural language processing applied to mental illness detection: a narrative review. NPJ Digit Med.

[3] Depression. World Health Organization.

[4] Evans-Lacko S, Aguilar-Gaxiola S, Al-Hamzawi A, Alonso J, Benjet C, Bruffaerts R, Chiu WT, Florescu S, de Girolamo G, Gureje O, Haro JM, He Y, Hu C, Karam EG, Kawakami N, Lee S, Lund C, Kovess-Masfety V, Levinson D, Navarro-Mateu F, Pennell BE, Sampson NA, Scott KM, Tachimori H, Ten Have M, Viana MC, Williams DR, Wojtyniak BJ, Zarkov Z, Kessler RC, Chatterji S, Thornicroft G (2018). Socio-economic variations in the mental health treatment gap for people with anxiety, mood, and substance use disorders: results from the WHO World Mental Health (WMH) surveys. Psychol Med.

[5] Yang K, Zhang T, Ananiadou S (2022). A mental state knowledge–aware and contrastive network for early stress and depression detection on social media. Inf Process Manage.

[6] Motrico E, Mateus V, Bina R, Felice E, Bramante A, Kalcev G, Mauri M (2020). Good Practices in perinatal mental health during the COVID-19 pandemic: a report from task-force RISEUP-PPD COVID-19. Clínica y Salud.

[7] Coronavirus and depression in adults, Great Britain. Office for National Statistics.

[8] Sheehan DV (2004). Depression: underdiagnosed, undertreated, underappreciated. Manag Care.

[9] Thombs BD, Kwakkenbos L, Levis AW, Benedetti A (2018). Addressing overestimation of the prevalence of depression based on self-report screening questionnaires. CMAJ.

[10] Lin H, Jia J, Nie L, Shen G, Chua T (2016). What does social media say about your stress?. Proceedings of the 25th International Joint Conference on Artificial Intelligence.

[11] Zhang T, Yang K, Ji S, Ananiadou S (2023). Emotion fusion for mental illness detection from social media: a survey. Inf Fusion.

[12] Zhang T, Yang K, Alhuzali H, Liu B, Ananiadou S (2023). PHQ-aware depressive symptoms identification with similarity contrastive learning on social media. Inf Process Manag.

[13] Issaka B, Aidoo EA, Wood SF, Mohammed F (2024). "Anxiety is not cute" analysis of twitter users' discourses on romanticizing mental illness. BMC Psychiatry.

[14] De Choudhury M (2013). Role of social media in tackling challenges in mental health. Proceedings of the 2nd international workshop on Socially-aware multimedia.

[15] Reece AG, Reagan AJ, Lix KL, Dodds PS, Danforth CM, Langer EJ (2017). Forecasting the onset and course of mental illness with Twitter data. Sci Rep.

[16] Chancellor S, De Choudhury M (2020). Methods in predictive techniques for mental health status on social media: a critical review. NPJ Digit Med.

[17] Ríssola EA, Losada DE, Crestani F (2021). A survey of computational methods for online mental state assessment on social media. ACM Trans Comput Healthcare.

[18] Amanat A, Rizwan M, Javed AR, Abdelhaq M, Alsaqour R, Pandya S, Uddin M (2022). Deep learning for depression detection from textual data. Electronics.

[19] Ghosh S, Ekbal A, Bhattacharyya P (2021). A multitask framework to detect depression, sentiment and multi-label emotion from suicide notes. Cogn Comput.

[20] Yoo M, Lee S, Ha T (2019). Semantic network analysis for understanding user experiences of bipolar and depressive disorders on Reddit. Inf Process Manag.

[21] Aragón ME, López Monroy AP, González-Gurrola L, Montes M (2019). Detecting depression in social media using fine-grained emotions. Proceedings of the 2019 Conference of the North American Chapter of the Association for Computational Linguistics: Human Language Technologies.

[22] Shickel B, Siegel S, Heesacker M, Benton S, Rashidi P (2020). Automatic detection and classification of cognitive distortions in mental health text. Proceedings of the 20th International Conference on Bioinformatics and Bioengineering.

[23] Settanni M, Marengo D (2015). Sharing feelings online: studying emotional well-being via automated text analysis of Facebook posts. Front Psychol.

[24] Cavazos-Rehg PA, Krauss MJ, Sowles S, Connolly S, Rosas C, Bharadwaj M, Bierut LJ (2016). A content analysis of depression-related Tweets. Comput Human Behav.

[25] Park J, Lee DS, Shablack H, Verduyn P, Deldin P, Ybarra O, Jonides J, Kross E (2016). When perceptions defy reality: the relationships between depression and actual and perceived Facebook social support. J Affect Disord.

[26] Golbeck J, Robles C, Turner K (2011). Predicting personality with social media. Proceedings of the 2011 Conference on Human Factors in Computing Systems.

[27] Tadesse MM, Lin H, Xu B, Yang L (2018). Personality predictions based on user behavior on the Facebook social media platform. IEEE Access.

[28] Klein DN, Kotov R, Bufferd SJ (2011). Personality and depression: explanatory models and review of the evidence. Annu Rev Clin Psychol.

[29] Hakulinen C, Elovainio M, Pulkki-Råback L, Virtanen M, Kivimäki M, Jokela M (2015). Personality and depressive symptoms: individual participant meta-analysis of 10 cohort studies. Depress Anxiety.

[30] Nguyen T, Yates A, Zirikly A, Desmet B, Cohan A (2022). Improving the generalizability of depression detection by leveraging clinical questionnaires. Proceedings of the 60th Annual Meeting of the Association for Computational Linguistics.

[31] Unlu A, Truong S, Tammi T, Lohiniva AL (2023). Exploring political mistrust in pandemic risk communication: mixed-method study using social media data analysis. J Med Internet Res.

[32] Kelman HC (1958). Compliance, identification, and internalization three processes of attitude change. J Conflict Resolut.

[33] (2017). About mental illness. Government of Canada.

[34] Neumann R, Strack F (2000). "Mood contagion": the automatic transfer of mood between persons. J Pers Soc Psychol.

[35] Gleick ME (2012). The Information: A History, a Theory, a Flood.

[36] (2020). Mental health myths and facts. MentalHealth.gov.

[37] Murarka A, Radhakrishnan B, Ravichandran S (2021). Classification of mental illnesses on social media using RoBERTa. Proceedings of the 12th International Workshop on Health Text Mining and Information Analysis.

[38] Cui B, Wang J, Lin H, Zhang Y, Yang L, Xu B (2022). Emotion-based reinforcement attention network for depression detection on social media: algorithm development and validation. JMIR Med Inform.

[39] Ghosal S, Jain A (2023). Depression and suicide risk detection on social media using fastText embedding and XGBoost classifier. Procedia Comput Sci.

[40] Kosinski M, Matz SC, Gosling SD, Popov V, Stillwell D (2015). Facebook as a research tool for the social sciences: opportunities, challenges, ethical considerations, and practical guidelines. Am Psychol.

[41] Mehmood A, Natgunanathan I, Xiang Y, Hua G, Guo S (2016). Protection of big data privacy. IEEE Access.

[42] Goldberg LR, Johnson JA, Eber HW, Hogan R, Ashton MC, Cloninger CR, Gough HG (2006). The international personality item pool and the future of public-domain personality measures. J Res Pers.

[43] Radloff LS (1977). The CES-D scale: a self-report depression scale for research in the general population. Appl Psychol Meas.

[44] Lewinsohn PM, Seeley JR, Roberts RE, Allen NB (1997). Center for Epidemiologic Studies Depression scale (CES-D) as a screening instrument for depression among community-residing older adults. Psychol Aging.

[45] Julian LJ, Gregorich SE, Tonner C, Yazdany J, Trupin L, Criswell LA, Yelin E, Katz PP (2011). Using the Center for Epidemiologic Studies Depression Scale to screen for depression in systemic lupus erythematosus. Arthritis Care Res (Hoboken).

[46] Bollen J, Gonçalves B, Ruan G, Mao H (2011). Happiness is assortative in online social networks. Artif Life.

[47] Mohammad SM (2018). Word affect intensities. Proceedings of the 11th Edition of the Language Resources and Evaluation Conference.

[48] Morinaga S, Yamanishi K, Tateishi K, Fukushima T (2002). Mining product reputations on the web. Proceedings of the 8th ACM SIGKDD International Conference on Knowledge Discovery and Data Mining.

[49] Rajaraman A, Ullman JD (2011). Mining of Massive Datasets.

[50] Bagby RM, Psych C, Quilty LC, Ryder AC (2008). Personality and depression. Can J Psychiatry.

[51] Ghosh S, Ekbal A, Bhattacharyya P (2023). VAD-assisted multitask transformer framework for emotion recognition and intensity prediction on suicide notes. Inf Process Manag.

[52] McCrae RR, Costa PT (1990). Personality in Adulthood: A Five-Factor Theory Perspective.

[53] McCrae RR, John OP (1992). An introduction to the five-factor model and its applications. J Pers.

[54] Preoţiuc-Pietro D, Eichstaedt J, Park G, Sap M, Smith L (2015). The role of personality, age, and gender in tweeting about mental illness. Proceedings of the 2nd Workshop on Computational Linguistics and Clinical Psychology: From Linguistic Signal to Clinical Reality.

[55] Yang X, McEwen R, Ong LR, Zihayat M (2020). A big data analytics framework for detecting user-level depression from social networks. Int J Inf Manage.

[56] Muñoz S, Iglesias CA (2022). A text classification approach to detect psychological stress combining a lexicon-based feature framework with distributional representations. Inf Process Manag.

[57] Yao X, Chen S, Yu G (2023). Effects of members’ response styles in an online depression community based on text mining and empirical analysis. Inf Process Manag.

[58] Devlin J, Chang MW, Lee K, Toutanova K (2021). BERT: pre-training of deep bidirectional transformers for language understanding. Proceedings of the 2021 Conference of the North American Chapter of the Association for Computational Linguistics: Human Language Technologies.

[59] Reimers N, Gurevych I (2019). Sentence-BERT: sentence embeddings using siamese BERT-networks. Proceedings of the 2019 Conference on Empirical Methods in Natural Language Processing and the 9th International Joint Conference on Natural Language Processing.

[60] Bowman SR, Angeli G, Potts C, Manning CD (2015). A large annotated corpus for learning natural language inference. Proceedings of the 2015 Conference on Empirical Methods in Natural Language Processing.

[61] Williams A, Nangia N, Bowman SR (2018). A broad-coverage challenge corpus for sentence understanding through inference. Proceedings of the 2018 Conference of the North American Chapter of the Association for Computational Linguistics: Human Language Technologies.

[62] Bagozzi RP, Dholakia UM (2002). Intentional social action in virtual communities. J Interact Mark.

[63] Slonim T (2014). Verbal behavior in individuals with generalized anxiety disorder and depressive disorders. City University of New York.

[64] (2020). Liwc2015: how it works. Linguistic Inquiry and Word Count.

[65] Coppersmith G, Dredze M, Harman C (2014). Quantifying mental health signals in Twitter. Proceedings of the 2014 Workshop on Computational Linguistics and Clinical Psychology: From Linguistic Signal to Clinical Reality.

[66] Coppersmith G, Dredze M, Harman C, Hollingshead K (2015). From ADHD to SAD: analyzing the language of mental health on twitter through self-reported diagnoses. Proceedings of the 2nd Workshop on Computational Linguistics and Clinical Psychology: From Linguistic Signal to Clinical Reality.

[67] De Choudhury M, Gamon M, Counts S, Horvitz E (2021). Predicting depression via social media. Proc Int AAAI Conf Web Soc Media.

[68] Chen X, Sykora MD, Jackson TW, Elayan S (2018). What about mood swings: identifying depression on Twitter with temporal measures of emotions. Proceedings of the 2018 Conference on World Wide Web.

[69] Marriwala N, Chaudhary D (2023). A hybrid model for depression detection using deep learning. Measur Sens.

[70] Breiman L (2001). Random forests. Machine Learn.

[71] Marerngsit S, Thammaboosadee S (2020). A two-stage text-to-emotion depressive disorder screening assistance based on contents from online community. Proceedings of the 8th International Conference on Electrical Engineering Congress.

[72] Wu W, Wu M, Yu K (2022). Climate and weather: inspecting depression detection via emotion recognition. Proceedings of the 2022 IEEE International Conference on Acoustics, Speech and Signal Processing.

[73] Sumner C, Byers A, Shearing M (2011). Determining personality traits and privacy concerns from Facebook activity. Black Hat Brief.

[74] Haque F, Nur RU, Jahan SA, Mahmud Z, Shah FM (2020). A transformer based approach to detect suicidal ideation using pre-trained language models. Proceedings of the 23rd International Conference on Computer and Information Technology.

[75] Jiang Z, Levitan SI, Zomick J, Hirschberg J (2020). Detection of mental health from Reddit via deep contextualized representations. Proceedings of the 2020 International Workshop on Health Text Mining and Information Analysis.

[76] Kleinman SS (2000). Social identification in a computer-mediated group for women in science and engineering. Sci Commun.

[77] McKenna KY, Bargh JA (2000). Plan 9 from cyberspace: the implications of the internet for personality and social psychology. Pers Soc Psychol Rev.

[78] Park M, McDonald D, Cha M (2021). Perception differences between the depressed and non-depressed users in Twitter. Proc Int AAAI Conf Web Soc Media.

[79] Mahmoudi N, Docherty P, Moscato P (2018). Deep neural networks understand investors better. Decis Support Syst.

