# Supplementary figures and images for: Psychological and Behavioral Insights From Social Media Users: Natural Language Processing–Based Quantitative Study on Mental Well-Being
Source: JMIR Form Res. 2025 Jan 20;9:e60286. doi: 10.2196/60286 (PMC11791453; doi:10.2196/60286)

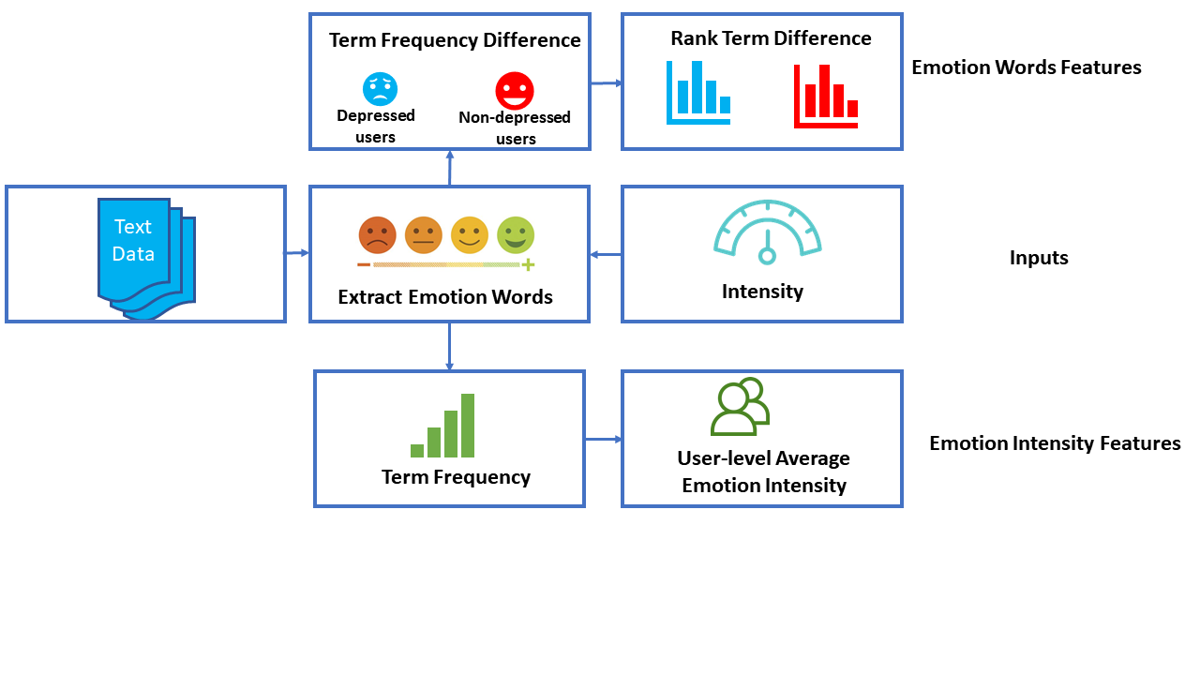

Supplement: Multimedia Appendix 1 [file formative_v9i1e60286_app1.png]

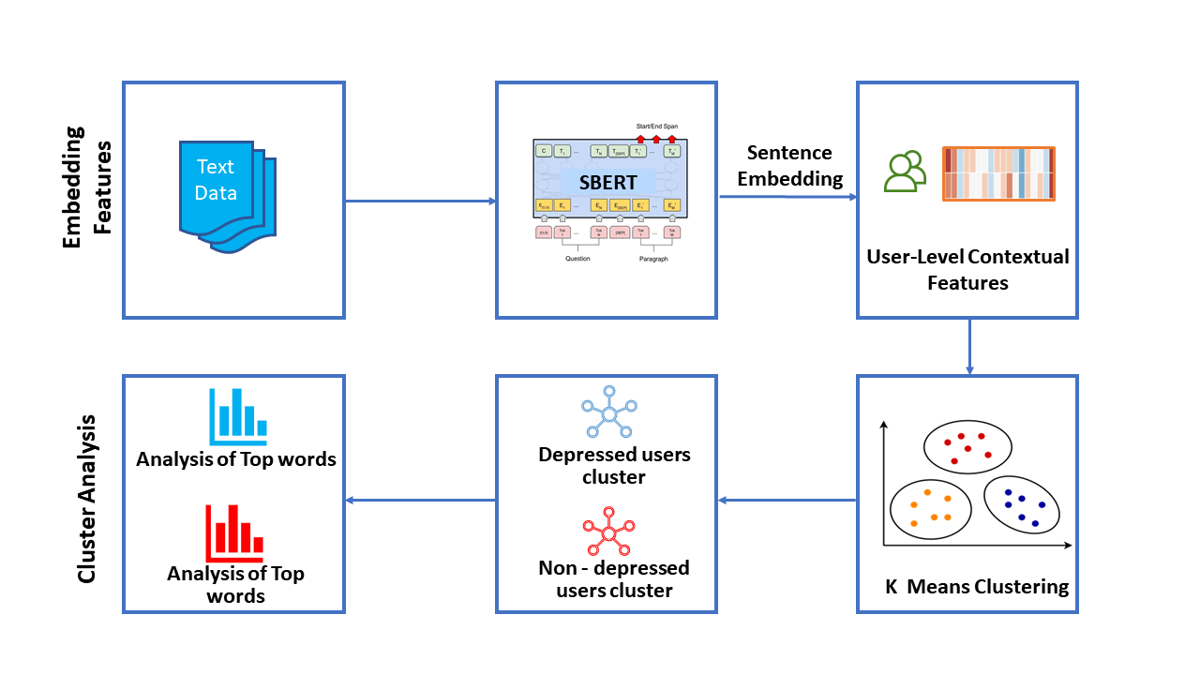

Supplement: Multimedia Appendix 3 [file formative_v9i1e60286_app3.png]

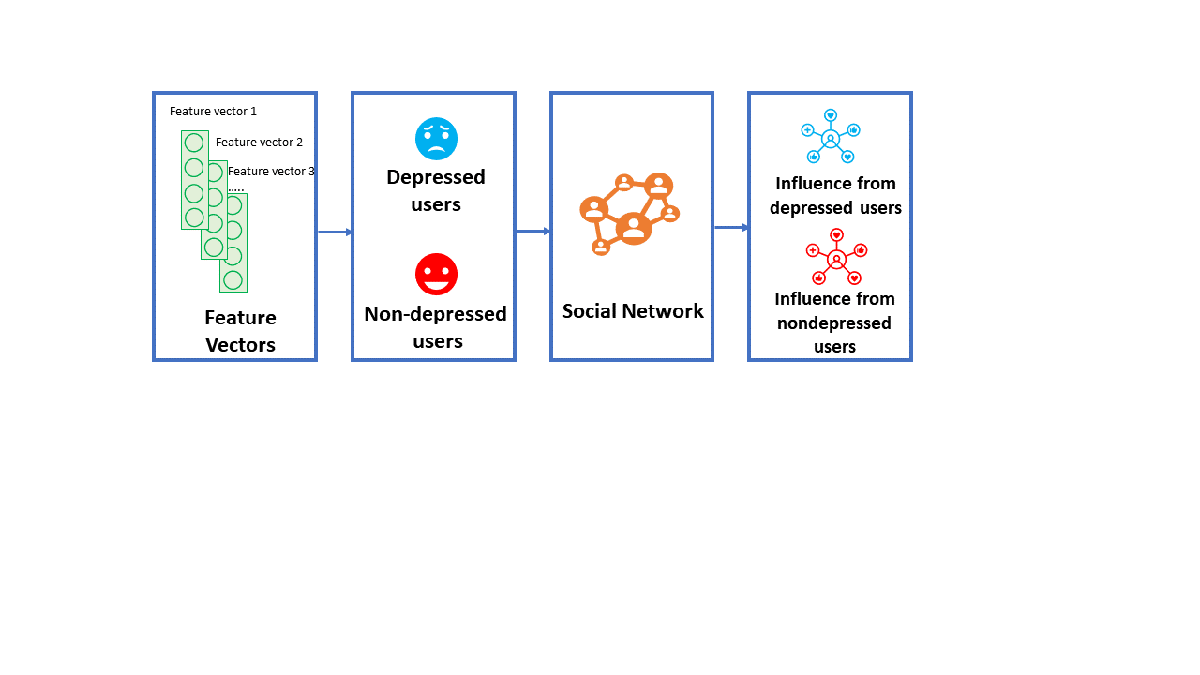

Supplement: Multimedia Appendix 4 [file formative_v9i1e60286_app4.png]

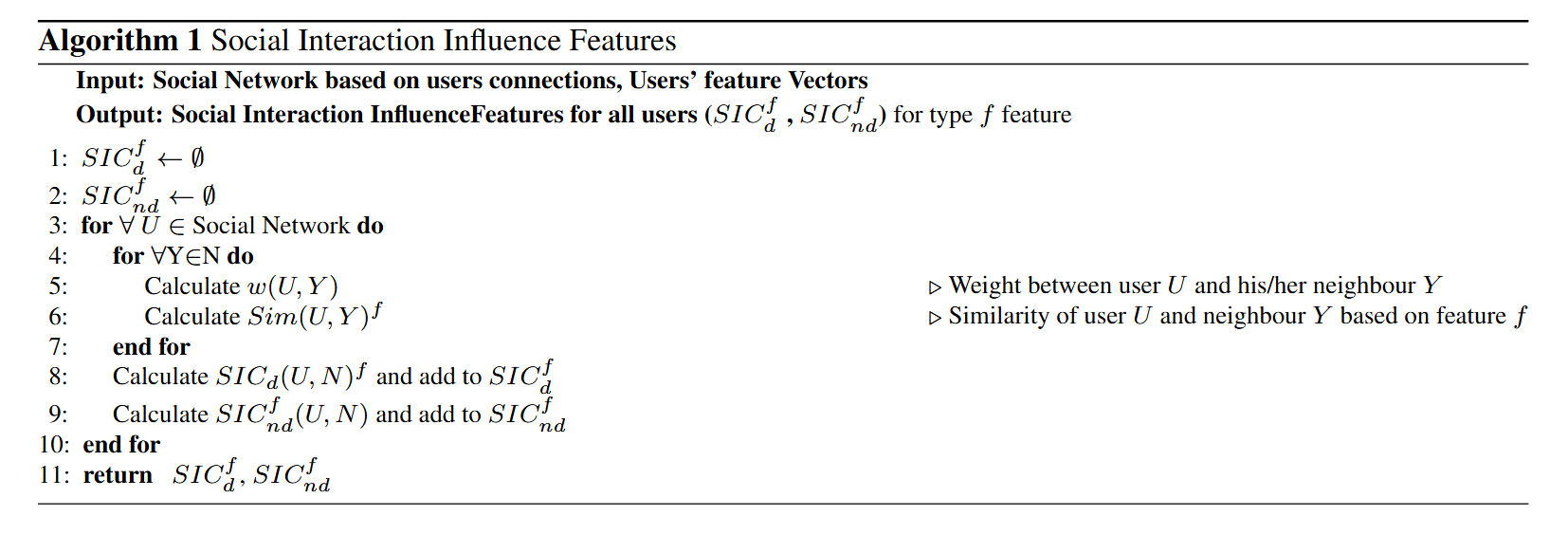

Supplement: Multimedia Appendix 5 [file formative_v9i1e60286_app5.png]

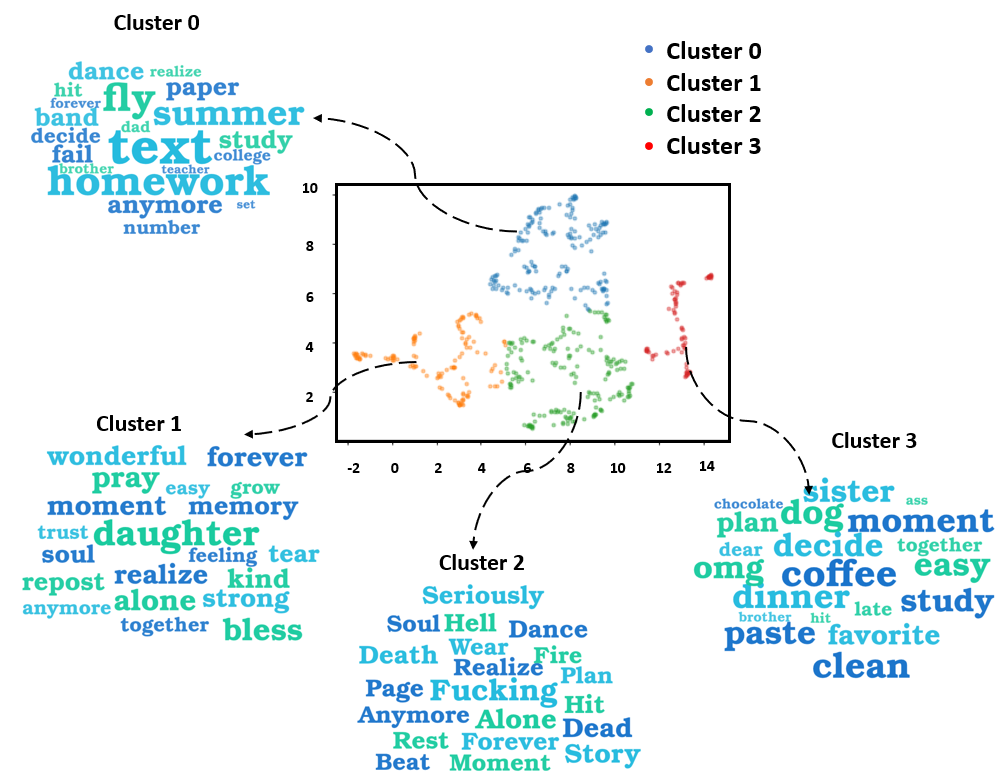

Supplement: Multimedia Appendix 6 [file formative_v9i1e60286_app6.png]

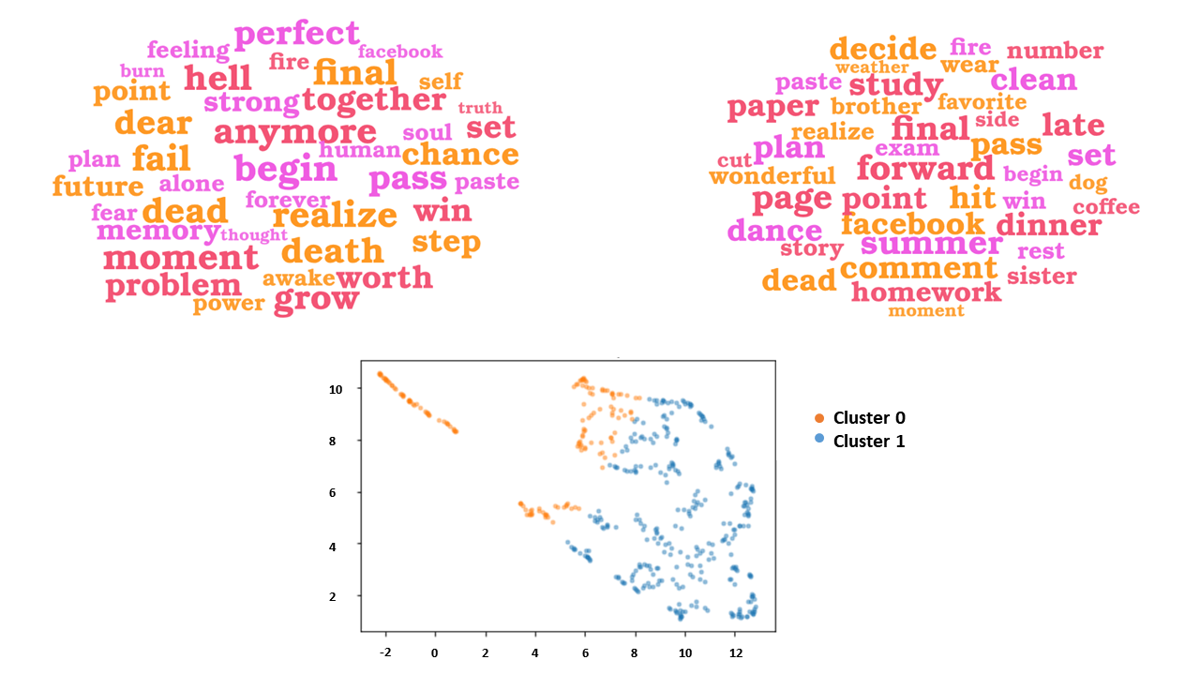

Supplement: Multimedia Appendix 7 [file formative_v9i1e60286_app7.png]

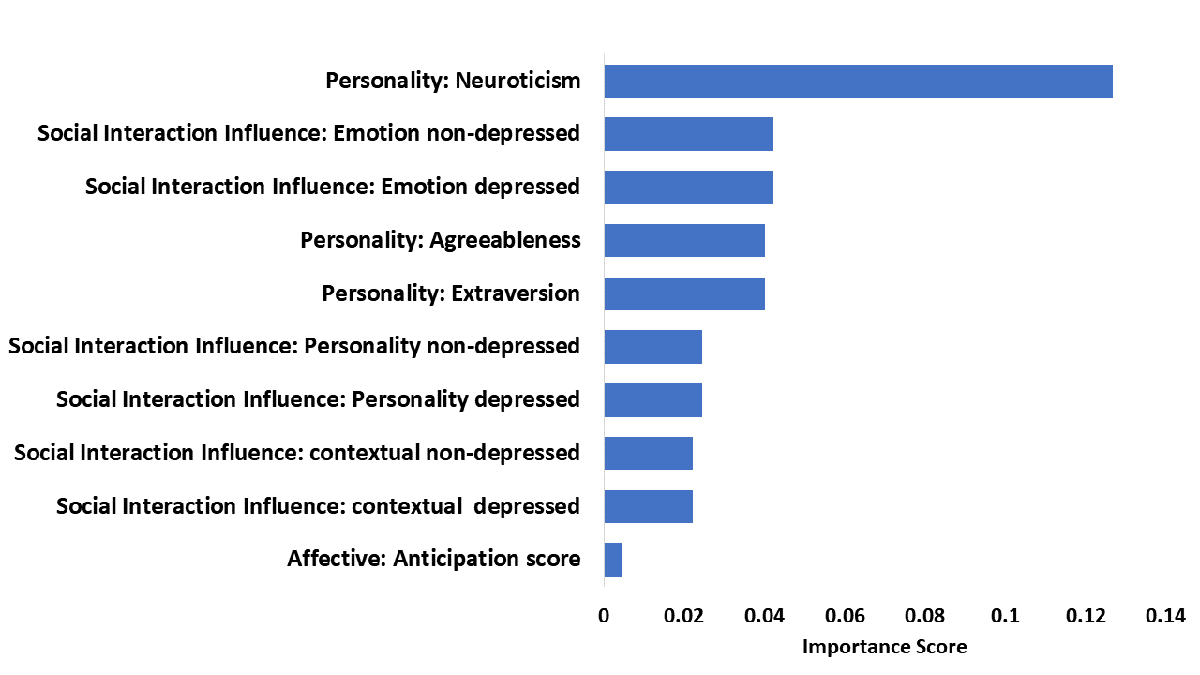

Supplement: Multimedia Appendix 8 [file formative_v9i1e60286_app8.png]
