# Supplementary material for: Psychological and Behavioral Insights From Social Media Users: Natural Language Processing–Based Quantitative Study on Mental Well-Being
Source: JMIR Form Res. 2025 Jan 20;9:e60286. doi: 10.2196/60286 (PMC11791453; doi:10.2196/60286)
Supplement: Multimedia Appendix 2 [file formative_v9i1e60286_app2.pdf]

Trait	Description	Low Score	High Score
**Neuroticism**	imagination, feelings, actions, ideas	practical, conventional, prefers routine	curious, wide range of interests, independent
**Extraversion**	competence, self-discipline, thoughtfulness, goal-driven	impulsive, careless, disorganized	hardworking, dependable, organized
**Openness**	sociability, assertiveness, emotional expression	quiet, reserved, withdrawn	outgoing, warm, seeks adventure
**Agreeableness**	cooperative, trustworthy, good-natured	critical, uncooperative, suspicious	helpful, trusting, empathetic
**Conscientiousness**	tendency toward unable emotions	calm, even-tempered, secure	anxious, unhappy, prone to negative emotions
